# HLA-DPA1 overexpression inhibits cancer progression, reduces resistance to cisplatin, and correlates with increased immune infiltration in lung adenocarcinoma

**DOI:** 10.18632/aging.205082

**Published:** 2023-10-27

**Authors:** Ke Shi, Qian-Yun Li, Yun-Qiang Zhang, Huan Huang, Dong-Xiao Ding, Wei-Min Luo, Jun Zhang, Qiang Guo

**Affiliations:** 1 Department of Thoracic Surgery, Beilun District People’s Hospital of Ningbo, Ningbo, China; 2 Department of Cardiothoracic Surgery, Taihe Hospital, Hubei University of Medicine, Shiyan, China; 3 The Fourth Affiliated Hospital, Zhejiang University School of Medicine, Yiwu, China; 4 Department of Thoracic Surgery, People’s Hospital of Dongxihu, Wuhan, China

**Keywords:** human leukocyte antigen-DP alpha 1, lung adenocarcinoma, prognosis, immune infiltration, The Cancer Genome Atlas

## Abstract

Purpose: Human Leukocyte Antigen-DP alpha 1 (HLA-DPA1) is a critical gene in antigen-presenting cells and plays a significant role in immune regulation. The objective of this study was to comprehensively analyze the roles of HLA-DPA1 and its association with lung adenocarcinoma (LUAD).

Methods: We utilized bioinformatics and conducted a meta-analysis to examine the roles of HLA-DPA1 expression on the progression and immunity of LUAD. We also performed CCK-8, wound healing, and Transwell assays to validate the functions of HLA-DPA1 in LUAD.

Results: HLA-DPA1 expression is downregulated in LUAD tissues and is associated with gender, race, age, smoking history, clinical stage, histological type, lymph node metastasis, and prognosis of patients with LUAD. HLA-DPA1 is involved in immune responses, leukocyte cell-cell adhesion, and antigen processing and presentation. Overexpression of HLA-DPA1 inhibits cancer cell proliferation, migration, and invasion while promoting cell sensitivity to cisplatin in A549 and A549/DDP cells. Additionally, overexpression of HLA-DPA1 correlates with tumor purity, stromal, immune, and ESTIMATE scores, the abundance of immune cells (B cells, CD8^+^ T cells, CD4^+^ T cells, macrophages, dendritic cells, and neutrophils), and immune cell markers (programmed cell death 1, cytotoxic T-lymphocyte-associated protein 4, and cluster of differentiation 8A).

Conclusions: Decreased HLA-DPA1 expression is associated with poor prognosis and immune infiltration in LUAD while HLA-DPA1 overexpression inhibits cancer cell proliferation and progression. Therefore, HLA-DPA1 shows potential as a prognostic biomarker and a therapeutic target for LUAD.

## INTRODUCTION

Lung cancer is a globally prevalent malignancy with one of the highest mortality rates among cancers [1]. Lung adenocarcinoma (LUAD) constitutes one of the most common subtypes of lung cancer [2, 3]. The immune system plays a pivotal role in tumorigenesis and cancer treatments. Immunotherapy has recently emerged as a promising therapeutic option for lung cancer [4–6]. For instance, programmed cell death protein 1 (PD-1) positive tumors lead to increased levels of T cells and programmed cell death ligand 1 (PD-L1) expression. Decreasing PD-1 expression helps control tumor growth, improves overall survival rates among cancer patients, and contributes to the reprogramming of tumor-associated lymph and myeloid cells [5]. A low-dose of apatinib, a small molecule tyrosine kinase inhibitor, alleviates hypoxia, increases cluster of differentiation (CD)8^+^ T cell infiltration, and reduces tumor-related macrophage recruitment in murine lung cancer models. Combining it with anti-PD-L1 effectively slows tumor growth, decreases cancer metastasis, and prolongs survival time. This combination can also enhance anticancer activity against advanced lung cancer [6]. Identifying new prognostic and immune-related targets for the progression of lung cancer represents a crucial need.

The major histocompatibility complex (MHC), DP alpha 1 (DPA1), also known as HLA-DPA1, located on chromosome 6p21.3, plays a critical role in the functioning of antigen-presenting cells, which are related to immune regulation. HLA-DPA1 is abnormally expressed in cancer tissues, contributing to cancer progression [7, 8]. For instance, downregulation of HLA-DPA1 expression is significantly associated with poor prognosis of adults with adrenocortical tumors, being an independent prognostic factor for these patients [7]. Similarly, HLA-DPA1 downregulation in multiple myeloma is correlated with disease-specific survival (DSS) of the affected patients [8]. However, the relationship between HLA-DPA1 expression, patient prognosis, and LUAD progression remains unclear. This study comprehensively explored the role of HLA-DPA1 in LUAD progression and the underlying pathways, aiming to identify a novel target for LUAD treatment.

## RESULTS

### Expression of HLA-DPA1 in LUAD tissues

**Figure 1:**
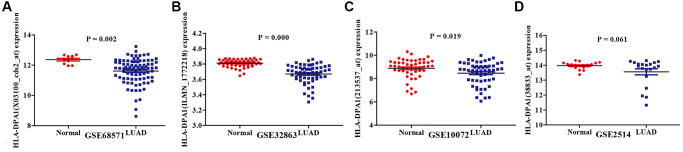
**HLA-DPA1 expression in lung adenocarcinoma tissues.** (**A**) GSE68751; (**B**) GSE32863; (**C**) GSE10072; (**D**) GSE2514.

**Figure 2:**
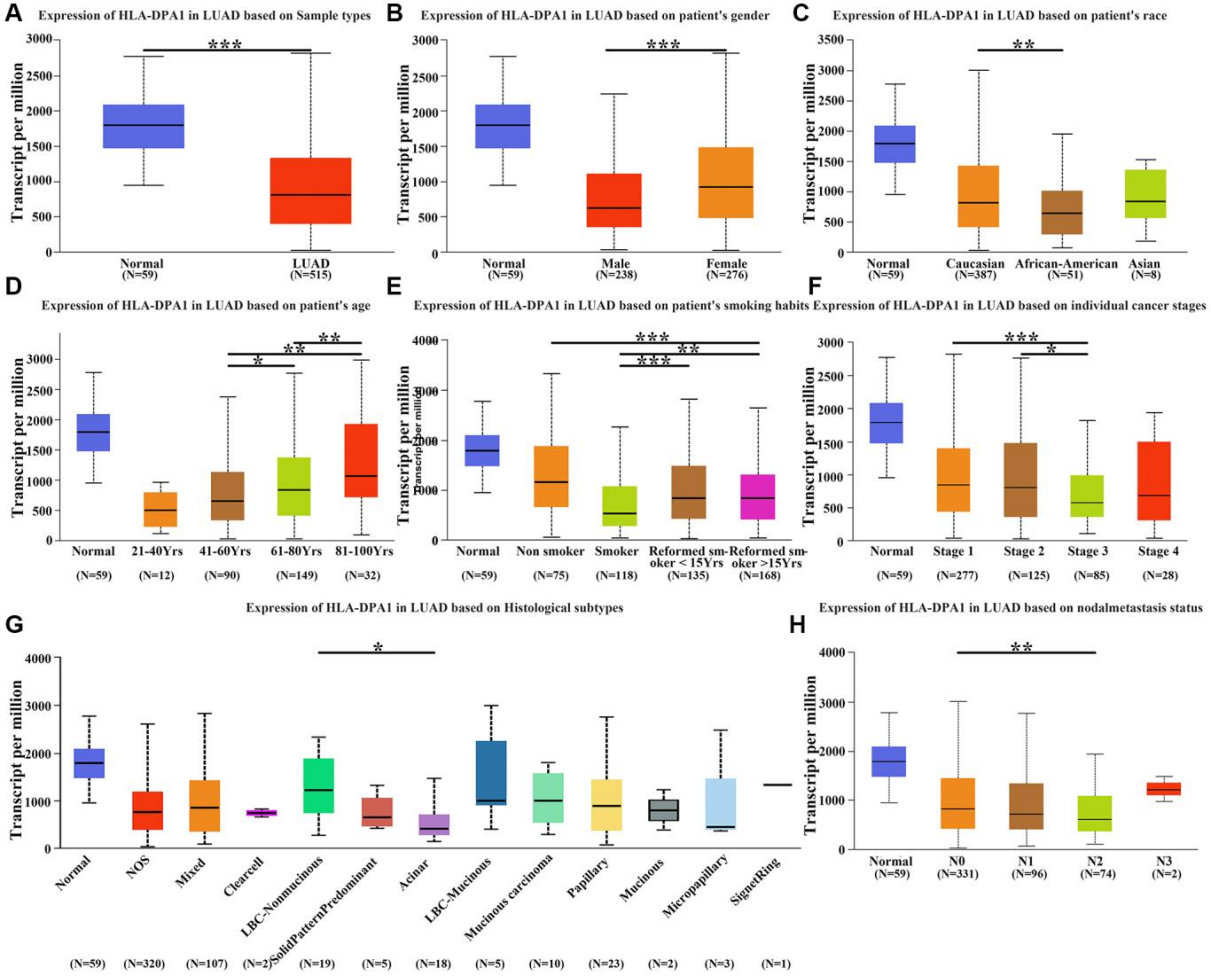
**The correlation between the expression of HLA-DPA1 and demographic indicators in LUAD.** (**A**) The expression of HLA-DPA1 in LUAD; its correlation with (**B**) Gender; (**C**) Race; (**D**) Age; (**E**) Smoking; (**F**) Clinical stage; (**G**) Tissue type; (**H**) Lymph node metastasis. Abbreviation: LUAD: lung adenocarcinoma; ^*^P < 0.05; ^**^P < 0.01; ^***^P < 0.001.

Based on data retrieved from the Gene Expression Omnibus (GEO) database, we found that HLA-DPA1 levels were significantly lower in LUAD tissues compared to normal tissue controls (Figure 1). The samples included 86 LUAD tissues vs. 10 normal tissues, 58 LUAD tissues vs. 58 normal tissues, 58 LUAD tissues vs. 49 normal tissues, and 20 LUAD tissues vs. 19 normal tissues from the GSE68751, GSE32863, GSE10072, and GSE2514 datasets, respectively (Figure 1A–1D). These trends were consistent when analyzing the data retrieved from the UALCAN database (Figure 2A).

### HLA-DPA1 expression correlates with the clinical indicators in patients with LUAD

Using data from the UALCAN database, we observed that decreased levels of HLA-DPA1 were associated with several demographic and clinical factors in patients with LUAD. These factors included gender, race, age, smoking history, clinical stage, histological subtype, and lymph node metastasis (Figure 2B–2H). Specifically, we found that HLA-DPA1 expression was lower in males compared to females, and in Caucasians compared to African Americans. Additionally, specific age ranges and smoking history were associated with decreased expression levels of HLA-DPA1. We also observed correlations between low HLA-DPA1 expression and the clinical stages, histological subtype, and lymph node metastasis categories in patients with LUAD (*P* < 0.05).

### HLA-DPA1 expression correlates with LUAD diagnosis and prognosis

**Figure 3:**
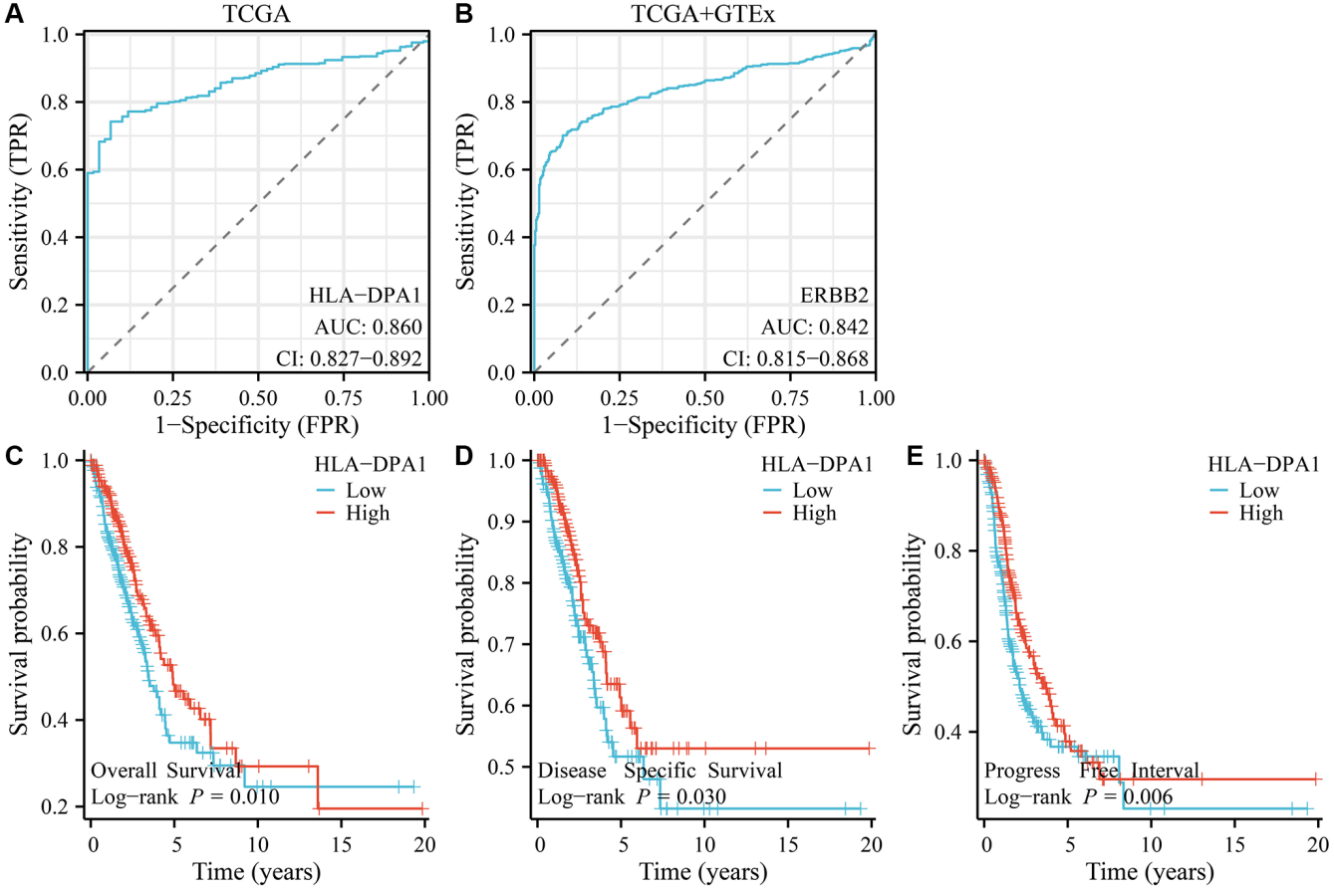
**The correlation between the expression of HLA-DPA1 and the diagnosis and prognosis of patients with LUAD.** (**A**, **B**) Diagnostic values of HLA-DPA1 in LUAD using ROC analysis; its correlation with (**C**) OS; (**D**) DSS; (**E**) PFI. Abbreviations: LUAD: lung adenocarcinoma; ROC: receiver operating characteristic; OS: overall survival; DSS: disease-specific survival; PFI: progress-free interval.

**Figure 4:**
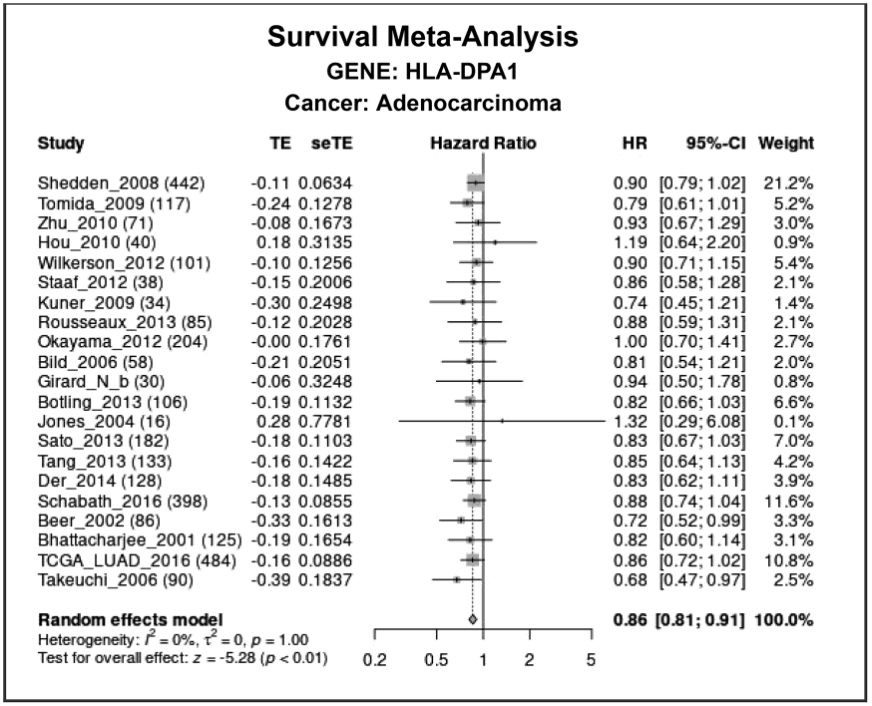
**The association between a decreased expression of HLA-DPA1 and poor prognosis of patients with LUAD using the LCE database.** Abbreviations: LUAD: lung adenocarcinoma; LCE: Lung Cancer Explorer.

Using data from both the Cancer Genome Atlas (TCGA) and XENA databases, we calculated that area under the curve for HLA-DPA1 in normal and cancer tissues were 0.86 and 0.842, respectively (Figure 3A, 3B). Our findings suggest that HLA-DPA1 has significant diagnostic significance. Moreover, our study revealed that a decrease in HLA-DPA1 expression was associated with poor prognosis, overall survival (OS), DSS, and progression-free interval (PFI), in patients with LUAD (Figure 3C–3E). Additionally, a meta-analysis of data within the Lung Cancer Explorer (LCE) database also demonstrated that decreased expression of HLA-DPA1 was related to poor OS for patients with LUAD (Figure 4).

### HLA-DPA1 roles and underlying mechanisms as revealed by Gene Ontology (GO) and Kyoto Encyclopedia of Genes and Genomes (KEGG) analyses

**Figure 5:**
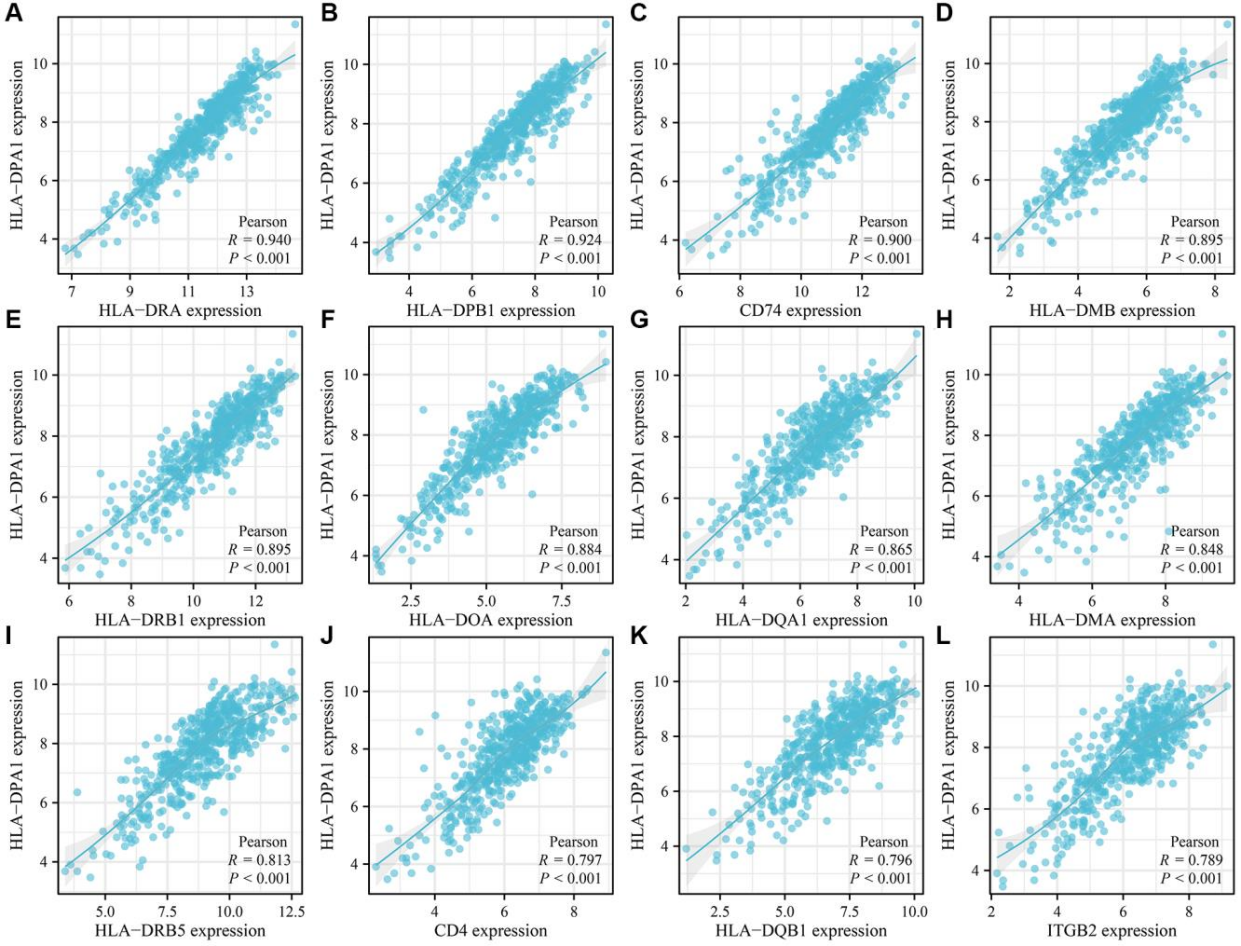
**Genes positively co-expressed with HLA-DPA1 in LUAD.** (**A**) HLA-DRA; (**B**) HLA-DPB1; (**C**) CD74; (**D**) HLA-DMB; (**E**) HLA-DRB1; (**F**) HLA-DOA; (**G**) HLA-DQA1; (**H**) HLA-DMA; (**I**) HLA-DRB5; (**J**) CD4; (**K**) HLA-DQB1; (**L**) ITGB2. Abbreviation: LUAD: lung adenocarcinoma.

Our analysis of LUAD tissues revealed 229 genes that were positively co-expressed with HLA-DPA1 based on correlation coefficients (Figure 5 and Supplementary Table 1). These co-expressed genes participate in various biological processes, including the MHC class II protein complex-mediated immune response, inflammatory response, positive regulation of T cell activation, cell surface functions, antigen processing and presentation, adaptive immune response, MHC class II receptor activity, as well as positive regulation of interferon-gamma production, tumor necrosis factor production, and T-cell mediated cytotoxicity. Furthermore, these genes are expressed in various signaling pathways, including cell adhesion molecules, Toll-like receptor, B cell receptor, Rap1, and chemokine signaling pathways, as well as Th1, Th2, and Th17 cell differentiation, natural killer cell-mediated cytotoxicity, and cytokine-cytokine receptor interaction, as revealed through GO and KEGG analyses (Supplementary Table 2 and Table 1). Utilizing the Tumor-Immune System Interaction Database (TISIDB), we revealed that HLA-DPA1 was involved in several biological processes, including the positive regulation of cytokine production, immune response, antigen processing and presentation, leukocyte cell-cell adhesion, T cell co-stimulation, proliferation, activation, and receptor signaling pathway, leukocyte proliferation, and presentation pathways, including cell adhesion molecules, antigen processing and presentation, and hematopoietic cell lineage pathways (Supplementary Table 3).

**Table 1:** Pathways associated with HLA-DPA1 co-expressed genes using KEGG analysis in the DAVID database.

**Term**	**Count**	***P***
hsa04145: Phagosome	30	2.85E-24
hsa05150: Staphylococcus aureus infection	24	9.59E-22
hsa05140: Leishmaniasis	21	9.12E-20
hsa05416: Viral myocarditis	19	3.94E-19
hsa05330: Allograft rejection	16	3.39E-18
hsa04514: Cell adhesion molecules	25	8.44E-18
hsa05152: Tuberculosis	26	1.73E-17
hsa04640: Hematopoietic cell lineage	21	1.98E-17
hsa04612: Antigen processing and presentation	19	7.10E-17
hsa05332: Graft-versus-host disease	15	7.80E-16
hsa05320: Autoimmune thyroid disease	16	1.11E-15
hsa04940: Type I diabetes mellitus	15	1.14E-15
hsa05145: Toxoplasmosis	20	4.26E-15
hsa05310: Asthma	13	1.22E-14
hsa04672: Intestinal immune network for IgA production	14	2.57E-13
hsa05323: Rheumatoid arthritis	17	5.81E-13
hsa05322: Systemic lupus erythematosus	19	2.03E-12
hsa05166: Human T-cell leukemia virus 1 infection	21	1.47E-10
hsa05321: Inflammatory bowel disease	13	2.25E-10
hsa04613: Neutrophil extracellular trap formation	19	5.89E-10
hsa04380: Osteoclast differentiation	16	9.20E-10
hsa04658: Th1 and Th2 cell differentiation	14	1.25E-09
hsa05169: Epstein-Barr virus infection	19	1.60E-09
hsa04659: Th17 cell differentiation	14	9.35E-09
hsa05164: Influenza A	15	3.43E-07
hsa05133: Pertussis	9	2.06E-05
hsa04062: Chemokine signaling pathway	13	3.91E-05
hsa05170: Human immunodeficiency virus 1 infection	13	1.02E-04
hsa04666: Fc gamma R-mediated phagocytosis	9	1.20E-04
hsa04611: Platelet activation	10	1.22E-04
hsa04650: Natural killer cell mediated cytotoxicity	10	1.38E-04
hsa04670: Leukocyte transendothelial migration	9	3.69E-04
hsa04664: Fc epsilon RI signaling pathway	7	6.10E-04
hsa05417: Lipid and atherosclerosis	11	0.001790779
hsa04610: Complement and coagulation cascades	7	0.002090384
hsa05168: Herpes simplex virus 1 infection	18	0.002223244
hsa05340: Primary immunodeficiency	5	0.002689423
hsa05167: Kaposi sarcoma-associated herpesvirus infection	10	0.003079788
hsa05171: Coronavirus disease - COVID-19	11	0.003122867
hsa05221: Acute myeloid leukemia	6	0.003684199
hsa05135: Yersinia infection	8	0.005261833
hsa04060: Cytokine-cytokine receptor interaction	12	0.0058967
hsa05134: Legionellosis	5	0.011492318
hsa03250: Viral life cycle - HIV-1	5	0.016148607
hsa04620: Toll-like receptor signaling pathway	6	0.022359401
hsa05163: Human cytomegalovirus infection	9	0.02325965
hsa04662: B cell receptor signaling pathway	5	0.037971909
hsa05144: Malaria	4	0.041906079
hsa04015: Rap1 signaling pathway	8	0.044169696

Abbreviation: KEGG, Kyoto Encyclopedia of Genes and Genomes.

### HLA-DPA1 expression impacts cell proliferation, migration, and sensitivity to cisplatin in LUAD

**Figure 6:**
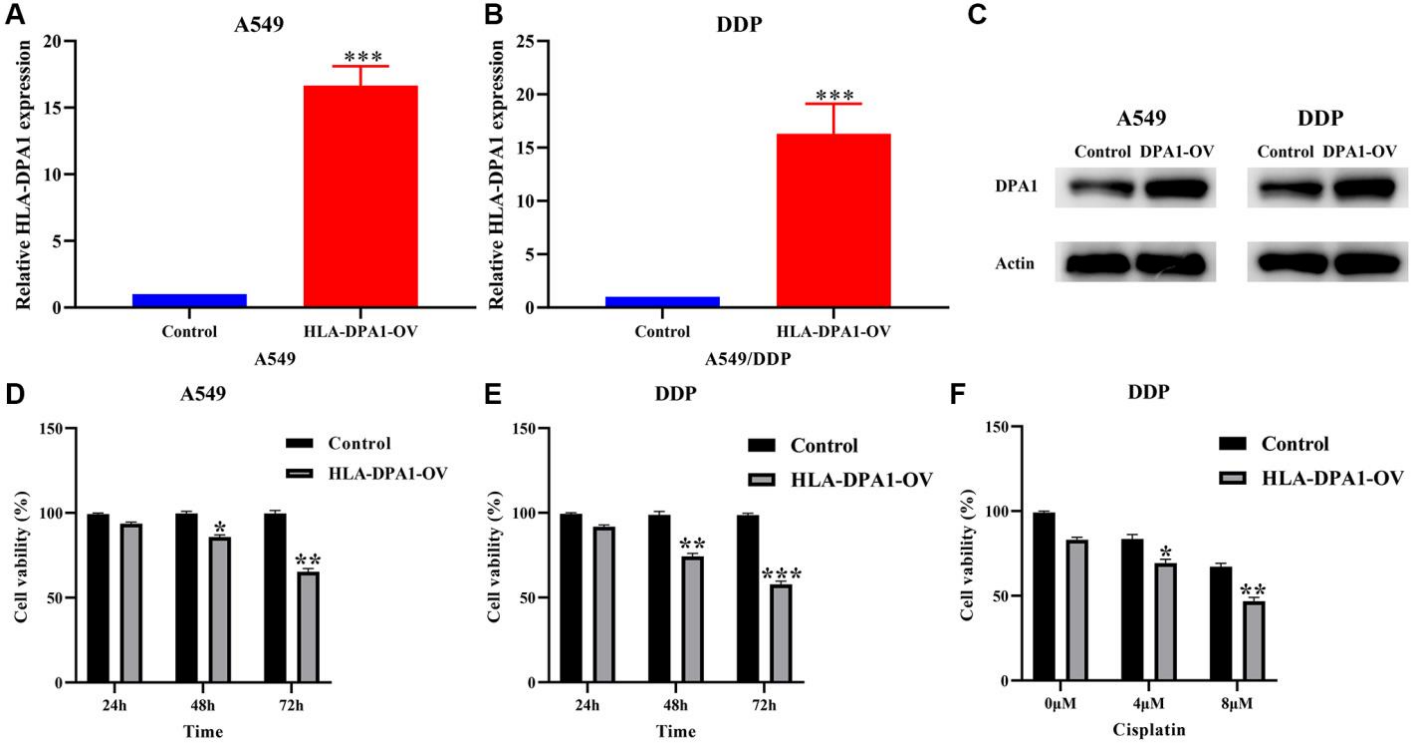
**HLA-DPA1 overexpression inhibits cancer cell proliferation and promotes cell sensitivity to cisplatin in LUAD.** (**A**–**C**) A549 and A549/DDP cell assays using RT-PCR and Western blotting (**D**, **E**) Assessment of A549 and A549/DDP cell proliferation; (**F**) A549/DDP cell sensitivity to cisplatin. Abbreviation: LUAD: lung adenocarcinoma.

**Figure 7:**
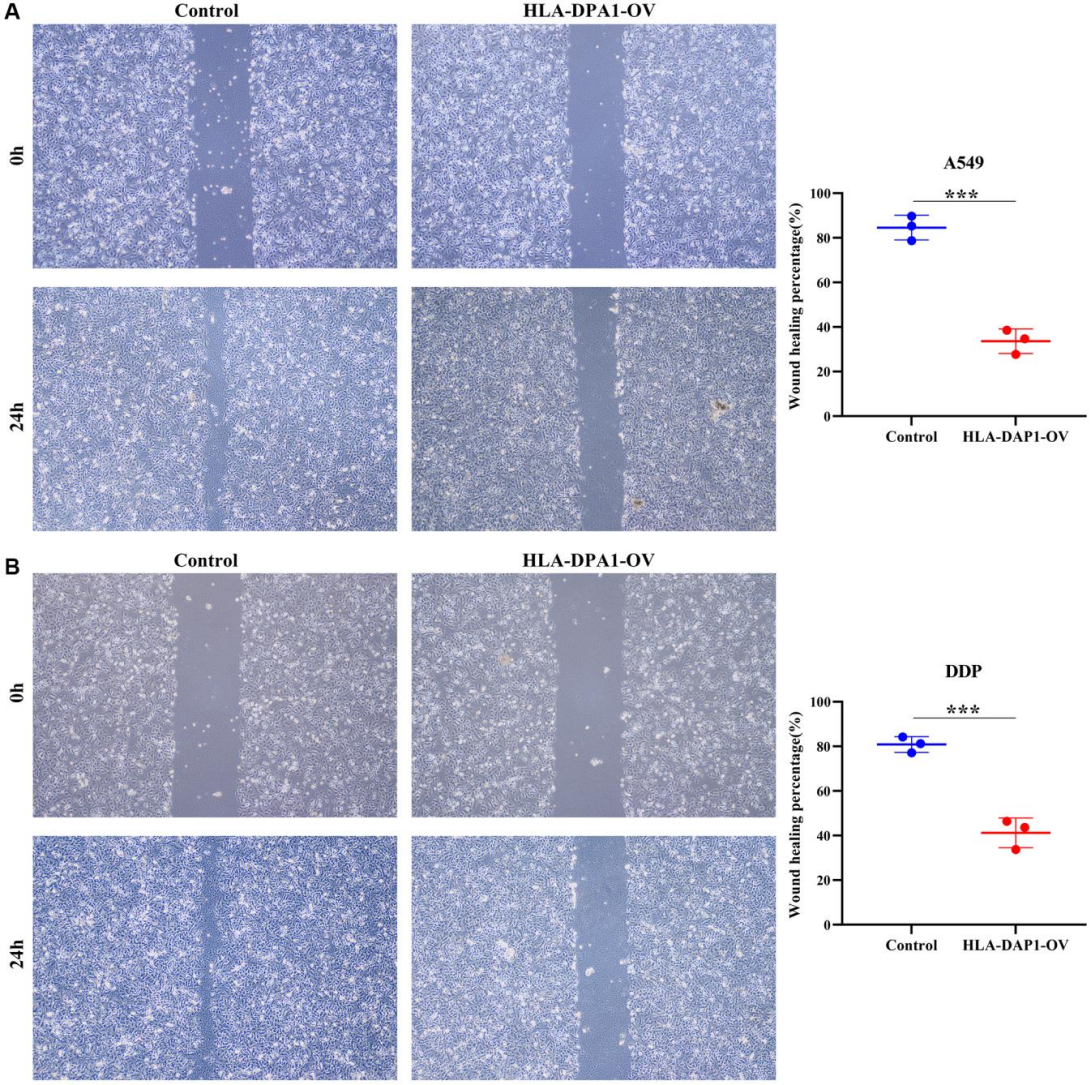
**HLA-DPA1 overexpression inhibits LUAD cell migration.** (**A**) A549; (**B**) A549/DDP. Abbreviation: LUAD: lung adenocarcinoma.

**Figure 8:**
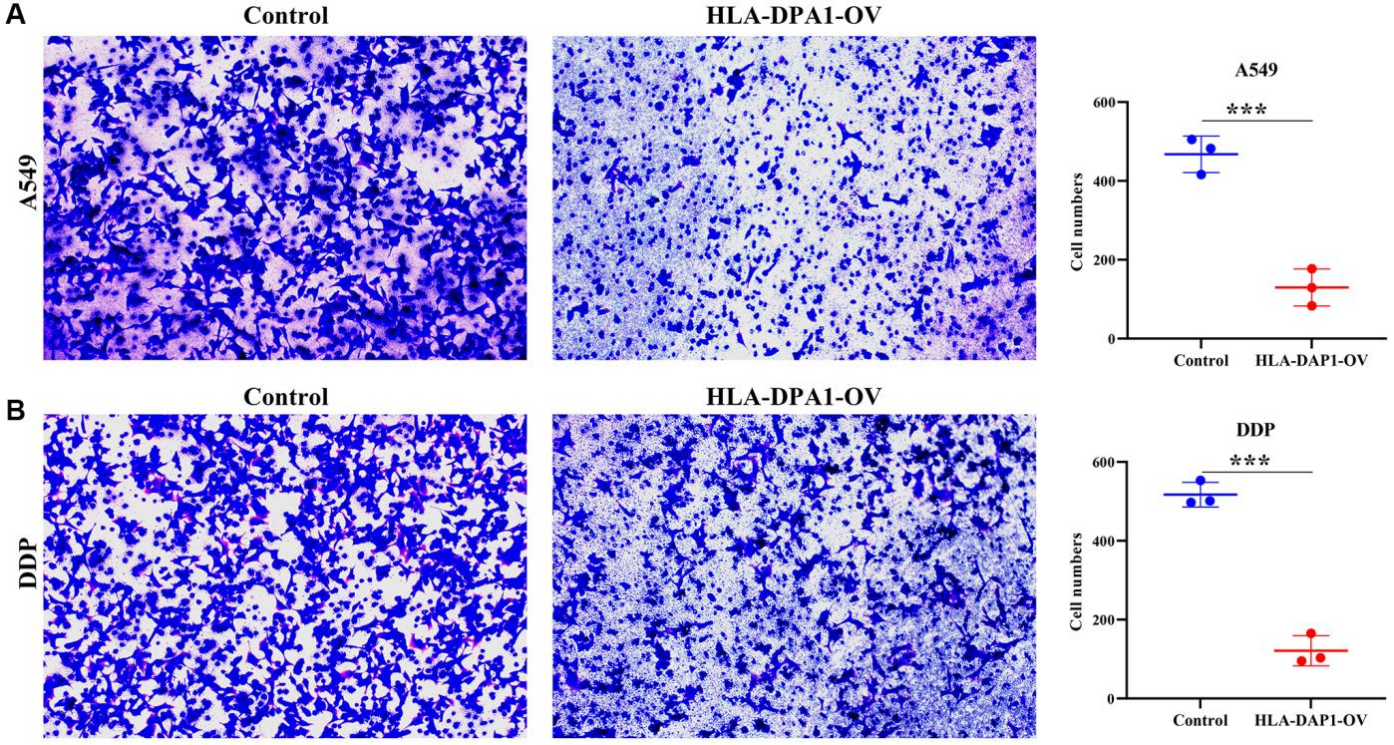
**HLA-DPA1 overexpression inhibits LUAD cell invasion.** (**A**) A549; (**B**) A549/DDP. Abbreviation: LUAD: lung adenocarcinoma.

HLA-DPA1 expression levels were significantly increased in LUAD cells compared to healthy cells, as confirmed by both Western blotting and polymerase chain reaction (PCR) (Figure 6A–6C). HLA-DPA1 overexpression resulted in a significant reduction in A549 and A549/DDP cell proliferation at both 48 h and 72 h, as demonstrated by CCK-8 assay (Figure 6D, 6E). Additionally, it increased the LUAD cell sensitivity to cisplatin, as depicted in Figure 6F. Finally, Transwell and wound healing assays revealed that HLA-DPA1 overexpression impacts the migration and invasion abilities of A549 and A549/DDP cells (Figures 7 and 8).

### HLA-DPA1 expression and LUAD immune microenvironment

**Figure 9:**
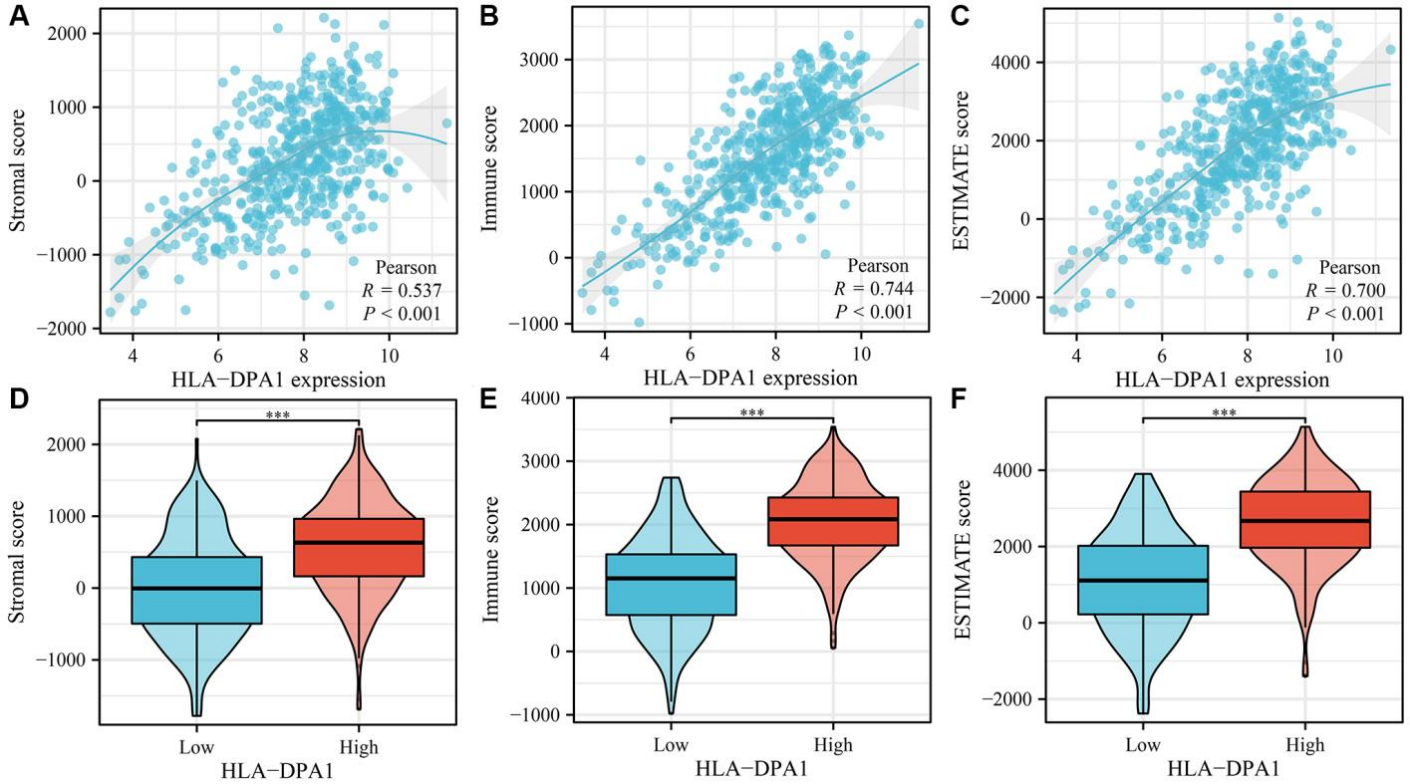
**The correlation between the HLA-DPA1 overexpression and stromal, immune, and ESTIMATE scores in LUAD.** (**A**–**C**) Correlation analysis with stromal, immune, and ESTIMATE scores; (**D**–**F**) Stromal, immune, and ESTIMATE scores in high- and low-HLA-DPA1 expression groups. Abbreviation: LUAD: lung adenocarcinoma.

**Figure 10:**
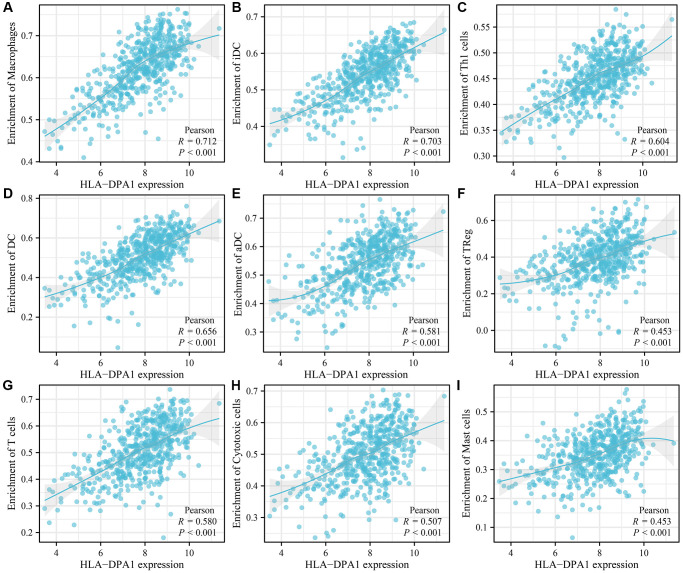
**The correlation between the HLA-DPA1 overexpression and immune cell levels in LUAD using data from The Cancer Genome Atlas.** (**A**) Macrophages; (**B**) iDC; (**C**) Th1 cells; (**D**) DC; (**E**) aDC; (**F**) TReg; (**G**) T cells; (**H**) Cytotoxic cells; (**I**) Mast cells. Abbreviations: LUAD: lung adenocarcinoma; DC: dendritic cells; Treg: regulatory T cells.

**Figure 11:**
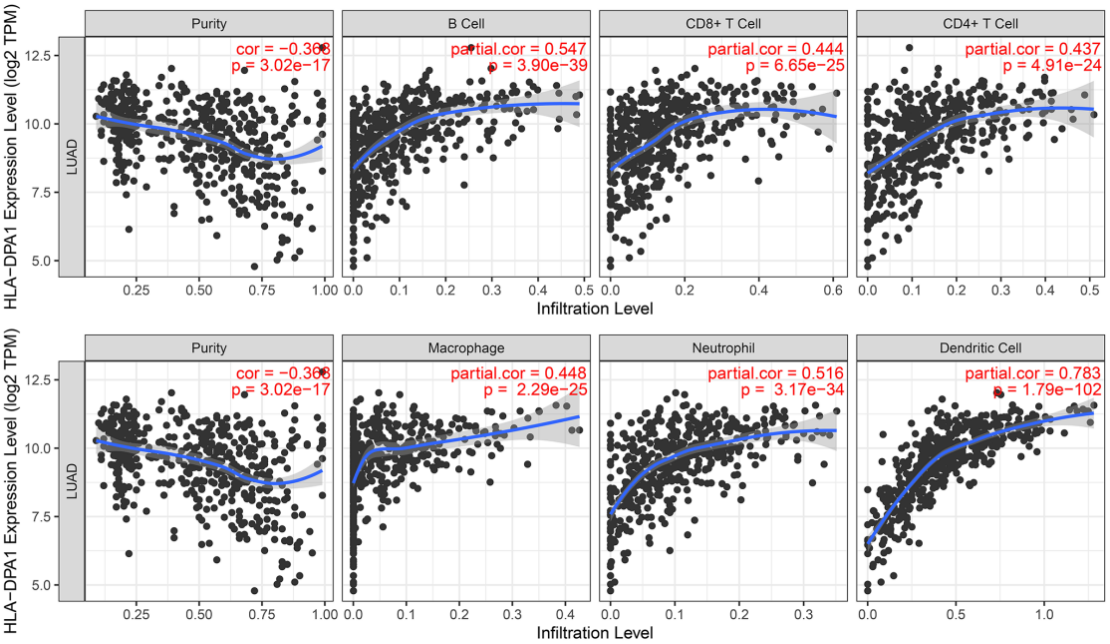
**The association between the HLA-DPA1 overexpression and tumor purity and immune cells in LUAD using the TIMER database.** Abbreviations: LUAD: lung adenocarcinoma; TIMER: Time Interval Medical Event Recorder.

In LUAD tissues, decreased HLA-DPA1 expression was positively correlated with stromal scores (r = 0.495), immune scores (r = 0.706), and ESTIMATE scores (r = 0.655) (Figure 9A–9C). Stromal, immune, and ESTIMATE scores differed significantly between the high- and low-HLA-DPA1 expression groups (Figure 9D–9F). Additionally, we observed a negative correlation between HLA-DPA1 expression and multiple immune cell types, including cytotoxic cells (r = 0.506885853), regulatory T cells (Treg) (r = 0.452668941), mast cells (r = 0.452567257), eosinophils (r = 0.450615318), T follicular helper cells (TFH) (r = 0.43046312), neutrophils (r = 0.396488996), plasmacytoid dendritic cells (pDCs) (r = 0.366524994), B cells (r = 0.337705922), CD8^+^ T cells (r = 0.321743937), T helper cells (r = 0.318979854), NK cells (r = 0.261549649), effector memory T cells (TEM) (r = 0.234554753), Th17 cells (r = 0.192560244), NK CD56dim cells (r = 0.19188812), central memory T cells (TCM) (r = 0.133514315), gamma delta T cells (TGD) (r = −0.144256151), and Th2 cells (r = −0.178080383) in LUAD (Figure 10 and Table 2). These differences in immune cell levels between HLA-DPA1 high-expression and low-expression groups are illustrated in Supplementary Figure 1. Using the Tumor Immune Estimation Resource (TIMER) database, we found a similar trend that a decreased HLA-DPA1 expression in LUAD was associated with reductions in immune cell populations, including B cells (r = 0.547), CD8^+^ T cells (r = 0.444), CD4^+^ T cells (r = 0.437), macrophages (r = 0.448), dendritic cells (r = 0.783), and neutrophils (r = 0.516) (Figure 11). Additionally, a significant inverse correlation was observed between HLA-DPA1 expression and tumor purity (r = −0.366).

**Table 2:** The association between the HLA-DPA1 overexpression and immune cells in LUAD.

**Gene**	**Immune cells**	**Coefficient**	***P***
HLA-DPA1	Macrophages	0.71192265	1.78742E-84
HLA-DPA1	iDC	0.702589054	2.18051E-81
HLA-DPA1	DC	0.655642609	1.57971E-67
HLA-DPA1	Th1 cells	0.603802207	7.53079E-55
HLA-DPA1	aDC	0.580758414	6.28424E-50
HLA-DPA1	T cells	0.580356886	7.59546E-50
HLA-DPA1	Cytotoxic cells	0.506885853	1.58675E-36
HLA-DPA1	TReg	0.452668941	1.37973E-28
HLA-DPA1	Mast cells	0.452567257	1.4236E-28
HLA-DPA1	Eosinophils	0.450615318	2.59103E-28
HLA-DPA1	TFH	0.43046312	1.00564E-25
HLA-DPA1	Neutrophils	0.396488996	9.79151E-22
HLA-DPA1	pDC	0.366524994	1.39729E-18
HLA-DPA1	B cells	0.337705922	7.62353E-16
HLA-DPA1	CD8 T cells	0.321743937	1.90451E-14
HLA-DPA1	T helper cells	0.318979854	3.26241E-14
HLA-DPA1	NK cells	0.261549649	7.01338E-10
HLA-DPA1	Tem	0.234554753	3.59003E-08
HLA-DPA1	Th17 cells	0.192560244	6.71982E-06
HLA-DPA1	NK CD56dim cells	0.19188812	7.245E-06
HLA-DPA1	Tcm	0.133514315	0.001893543
HLA-DPA1	NK CD56bright cells	0.011507791	0.789809688
HLA-DPA1	Tgd	−0.144256151	0.000782494
HLA-DPA1	Th2 cells	−0.178080383	3.20957E-05

Abbreviation: LUAD: lung adenocarcinoma.

### HLA-DPA1 expression was correlated with immune cell markers in LUAD

**Figure 12:**
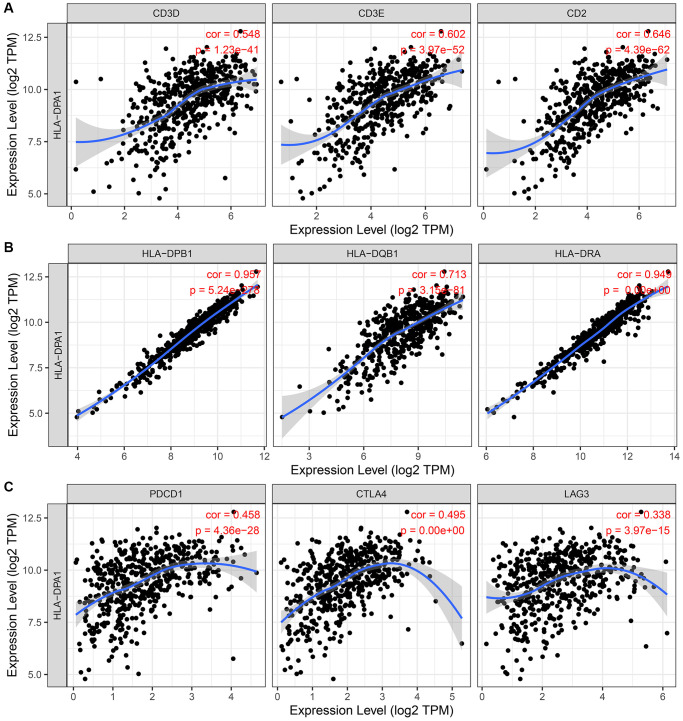
**The association between the HLA-DPA1 overexpression and immune cell markers in LUAD using the TIMER database.** (**A**) T cell markers; (**B**) Dendritic cell markers; (**C**) T cell exhaustion markers. Abbreviations: DC: dendritic cells; LUAD: lung adenocarcinoma.

**Figure 13:**
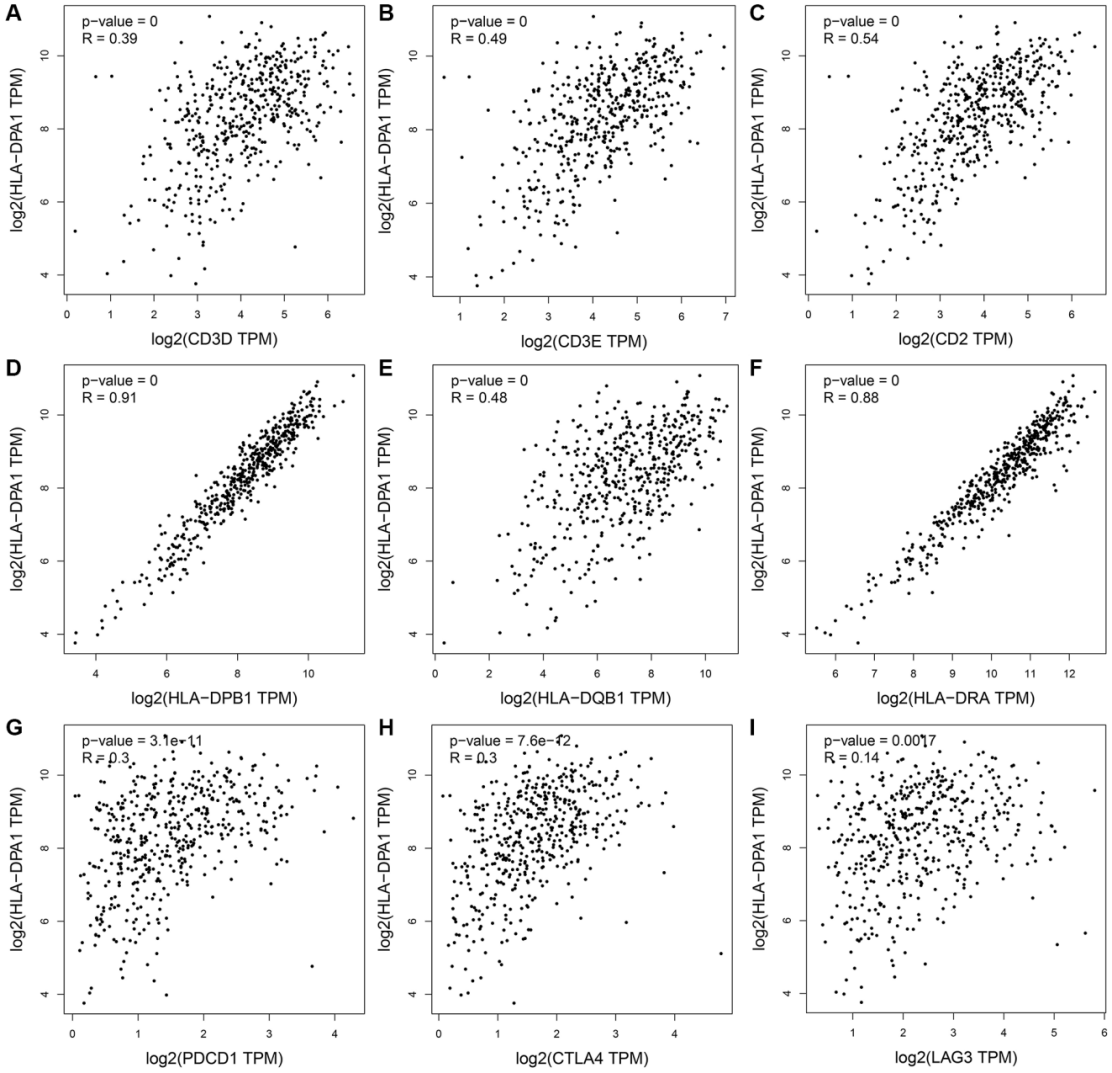
**The association between the HLA-DPA1 overexpression and immune cell markers in LUAD using the GEPIA database.** (**A**) CD3D; (**B**) CD3E; (**C**) CD2; (**D**) HLA-DPB1; (**E**) HLA-DQB1; (**F**) HLA-DRA; (**G**) PDCD1; (**H**) CTLA4; (**I**) LAG3. Abbreviation: LUAD: lung adenocarcinoma.

Using data from the TIMER database, we revealed a significant correlation between the decreased expression of HLA-DPA1 and T cell exhaustion as well as markers of various immune cell types, including CD8^+^ T cells, T cells, B cells, monocytes, tumor-associated macrophages, macrophages, neutrophils, natural killer cells, dendritic cells, T helper cells, and Treg (Figure 12 and Table 3). Furthermore, in the context of tumor purity, correlation trends remained consistent in LUAD (Table 4 and Supplementary Figure 2). We found similar correlations using Gene Expression Profiling Interactive Analysis (GEPIA) (Table 5 and Figure 13). Genes significantly associated with decreased HLA-DPA1 expression in LUAD tissues included those encoding various immune cell markers, such as CD8A, CD8B, CD3D, CD2, CD3E, CD19, CD79A, CD86, CSF1R, CCL2, CD68, IL10, IRF5, PTGS2, CD163, VSIG4, MS4A4A, CEACAM8, ITGAM, CCR7, IL21, STAT3, HLA-DPB1, HLA-DQB1, HLA-DRA, CD1C, NRP1, ITGAX, TBX21, TNF, STAT4, STAT1, IFNG, STAT6, STAT5A, IL13, FOXP3, CCR8, STAT5B, TGFB1, PDCD1, CTLA4, LAG3, and HAVCR2.

**Table 3:** The correlation between the HLA-DPA1 expression and the level of immune cell markers in LUAD.

**Gene**	**Coefficient**	***P***	**Gene**	**Coefficient**	***P***
CD8A	0.463860846	^***^	HLA-DPB1	0.957048805	^***^
CD8B	0.383594772	^***^	HLA-DQB1	0.713309359	^***^
CD3D	0.547637137	^***^	HLA-DRA	0.948978566	^***^
CD3E	0.602153884	^***^	CD1C	0.622583525	^***^
CD2	0.645701555	^***^	NRP1	0.260608494	^***^
CD19	0.328131252	^***^	ITGAX	0.590846161	^***^
CD79A	0.301133316	^***^	TBX21	0.497153269	^***^
CD86	0.717281395	^***^	STAT4	0.56603653	^***^
CSF1R	0.720382056	^***^	STAT1	0.349391568	^***^
CCL2	0.40749069	^***^	IFNG	0.313908839	^***^
CD68	0.601204653	^***^	TNF	0.43121415	^***^
IL10	0.528217991	^***^	GATA3	0.448382268	^***^
NOS2	0.024972061	0.572	STAT6	0.240980432	^***^
IRF5	0.48368983	^***^	STAT5A	0.627124519	^***^
PTGS2	−0.149620779	^***^	IL13	0.191836762	^***^
CD163	0.551646778	^***^	BCL6	0.093426945	^*^
VSIG4	0.615448587	^***^	IL21	0.234191463	^***^
MS4A4A	0.647014328	^***^	STAT3	0.115660054	^**^
CEACAM8	0.387397902	^***^	IL17A	0.156174024	^***^
ITGAM	0.693646404	^***^	FOXP3	0.557837581	^***^
CCR7	0.573222298	^***^	CCR8	0.564637826	^***^
KIR2DL1	0.080908061	0.067	STAT5B	0.255361072	^***^
KIR2DL3	0.141185802	^**^	TGFB1	0.47796272	^***^
KIR2DL4	0.097084787	^*^	PDCD1	0.458139335	^***^
KIR3DL1	0.109261553	^*^	CTLA4	0.4946446	^***^
KIR3DL2	0.195677599	^***^	LAG3	0.338210486	^***^
KIR3DL3	−0.018825523	0.670	HAVCR2	0.72004897	^***^
KIR2DS4	0.14515847	^***^	GZMB	0.193203672	^***^

Abbreviation: LUAD: lung adenocarcinoma; ^*^P < 0.05; ^**^P < 0.01; ^***^P < 0.001.

**Table 4:** The correlation between the HLA-DPA1 overexpression and immune cell markers in the context of tumor purity.

**Gene**	**Coefficient**	***P***	**Gene**	**Coefficient**	***P***
CD8A	0.361547657	^***^	HLA-DPB1	0.951444602	^***^
CD8B	0.295531118	^***^	HLA-DQB1	0.678456948	^***^
CD3D	0.44980375	^***^	HLA-DRA	0.943553019	^***^
CD3E	0.516992518	^***^	CD1C	0.589149871	^***^
CD2	0.57140071	^***^	NRP1	0.240701613	^***^
CD19	0.191985178	^***^	ITGAX	0.517238257	^***^
CD79A	0.163961649	^***^	TBX21	0.407921541	^***^
CD86	0.664542948	^***^	STAT4	0.493977846	^***^
CSF1R	0.669761973	^***^	STAT1	0.266515617	^***^
CCL2	0.324125987	^***^	IFNG	0.213157882	^***^
CD68	0.542372297	^***^	TNF	0.335546484	^***^
IL10	0.444583773	^***^	GATA3	0.348966526	^***^
NOS2	-0.057981416	0.199	STAT6	0.275594139	^***^
IRF5	0.425447924	^***^	STAT5A	0.560979818	^***^
PTGS2	-0.163392491	^***^	IL13	0.123975663	^**^
CD163	0.478418571	^***^	BCL6	0.082693089	0.067
VSIG4	0.566379418	^***^	IL21	0.161538758	^***^
MS4A4A	0.588284315	^***^	STAT3	0.127863377	^**^
CEACAM8	0.39364805	^***^	IL17A	0.08745736	0.052
ITGAM	0.647768352	^***^	FOXP3	0.474996557	^***^
CCR7	0.485288268	^***^	CCR8	0.487678859	^***^
KIR2DL1	0.017664227	0.696	STAT5B	0.24347394	^***^
KIR2DL3	0.062591704	0.165	TGFB1	0.417646704	^***^
KIR2DL4	0.011009074	0.807	PDCD1	0.356089872	^***^
KIR3DL1	0.042372557	0.348	CTLA4	0.384848895	^***^
KIR3DL2	0.115349466	^*^	LAG3	0.238139944	^***^
KIR3DL3	-0.05426989	0.229	HAVCR2	0.668044203	^***^
KIR2DS4	0.076415879	0.090	GZMB	0.058566398	0.194

Abbreviation: LUAD: lung adenocarcinoma; ^*^P < 0.05; ^**^P < 0.01; ^***^P < 0.001.

**Table 5:** The association between the HLA-DPA1 overexpression and immune cell markers in LUAD tissues from the GEPIA database.

**Gene**	**Coefficient**	***P***	**Gene**	**Coefficient**	***P***
CD8A	0.35	^***^	CD1C	0.42	^***^
CD8B	0.23	^***^	NRP1	0.27	^***^
CD3D	0.39	^***^	ITGAX	0.40	^***^
CD3E	0.49	^***^	TBX21	0.11	^*^
CD2	0.54	^***^	STAT4	0.41	^***^
CD19	0.24	^***^	STAT1	0.29	^***^
CD79A	0.20	^***^	IFNG	0.24	^***^
CD86	0.62	^***^	TNF	0.3	^***^
CSF1R	0.59	^***^	GATA3	0.0024	0.96
CCL2	0.23	^***^	STAT6	0.27	^***^
CD68	0.49	^***^	STAT5A	0.56	^***^
IL10	0.44	^***^	IL13	0.21	^***^
IRF5	0.32	^***^	BCL6	0.14	0.068
PTGS2	-0.14	^**^	IL21	0.29	^***^
CD163	0.32	^***^	STAT3	0.17	^***^
VSIG4	0.46	^***^	IL17A	0.12	0.072
MS4A4A	0.49	^***^	FOXP3	0.48	^***^
CEACAM8	0.25	^***^	CCR8	0.48	^***^
ITGAM	0.54	^***^	STAT5B	0.25	^***^
CCR7	0.43	^***^	TGFB1	0.32	^***^
KIR3DL2	-0.061	0.18	PDCD1	0.30	^***^
HLA-DPB1	0.91	^***^	CTLA4	0.30	^***^
HLA-DQB1	0.48	^***^	LAG3	0.14	^**^
HLA-DRA	0.88	^***^	HAVCR2	0.59	^***^

Abbreviation: LUAD: lung adenocarcinoma; ^*^P < 0.05; ^**^P < 0.01; ^***^P < 0.001.

## DISCUSSION

In recent years, LUAD emerged as a prevalent subtype of non-small-cell lung cancer due to its increasing incidence [9–11]. Genetic alterations substantially impact the progression of lung cancer and can be indicative of poor prognosis in patients with cancer. Notably, GALNT2 overexpression in LUAD tissues is associated with worse patient outcomes. *In vitro* experiments demonstrate that downregulation of GALNT2 suppresses cell proliferation, migration, and invasion, while its overexpression accelerates these processes and activates Notch/Hairy and Enhancer of Split 1/phosphatase and tensin homolog/phosphatidylinositol 3-kinase/protein kinase B signaling, inducing cancerous transformation [9]. Similarly, MRPL42 overexpression is observed in the early stages of LUAD. Knockdown of MRPL42 expression reduces cell proliferation and colony formation, induces cell cycle arrest, and inhibits migration and invasion of LUAD cells [10]. However, the relationship between HLA-DPA1 and LUAD remains unexplored. Our analysis revealed decreased HLA-DPA1 expression in LUAD tissues, associated with gender, race, age, smoking history, clinical stage, histological type, and lymph node metastasis of patients with LUAD. Furthermore, HLA-DPA1 expression has diagnostic value and is correlated with OS, DSS, and PFI of patients with LUAD. Our preliminary findings suggest that HLA-DPA1 may serve as a potential prognostic marker in this pathology.

DCs are vital for antigen presentation and cancer progression. They process foreign antigens and present them to CD4^+^ T cells, with activated DCs changing MHC class II antigen presentation mechanisms to CD4^+^ T cells [12]. PD-L1, expressed on tumor-related DCs in patients with lung cancer, binds to the B7.1 receptor. Blocking PD-L1 on DCs reduces its binding to B7.1, enhancing T cell activation and increasing OS in patients with non-small cell lung cancer receiving PD-L1 blocker therapy [13]. HLA-DPA1 is a marker of DCs, and HLA-DPA1 co-expressed genes are involved in various biological processes such as inflammatory response, positive regulation of T cell activation, antigen processing and presentation, adaptive immune response, and T cell-mediated cytotoxicity, among others. Our study further revealed that HLA-DPA1 overexpression could inhibit cell proliferation and migration, as well as promote cell sensitivity to cisplatin. These findings indicate that HLA-DPA1 is involved in LUAD progression as a tumor suppressor gene.

The relationship between immune cells, immune-related factors, and cancer progression has been well-established [14–18]. For instance, FAM83H overexpression inhibits infiltration of tumor-infiltrating lymphocytes (TILs) and anti-tumor activity, particularly that of CD8^+^ T cells. Its overexpression level inversely correlates with the expression levels of CD8A, CD8B, CD2, CD3D, and CD3E [14]. In colorectal cancer, increased sCD163 expression indicates poorer OS and DFS, with high sCD163 levels and monocytes significantly influencing DFS in these patients [16]. Our study establishes a link between the decreased expression of HLA-DPA1 and various immune cell subsets, such as Treg, mast cells, eosinophils, TFH, neutrophils, pDCs, B cells, CD8^+^ T cells, T helper cells, NK cells, Tem, Th17 cells, NK CD56dim cells, Tcm, Tgd, and Th2 cells. Additionally, we identified associations with the markers of overall immune response, such as CD8A and CD8B for CD8^+^ T cells, CD3D, CD3E, and CD2 for T cells, and CD19 and CD79A for B cells, as well as those of T cell exhaustion such as PDCD1, CTLA4, lymphocyte activation gene 3, hepatitis A virus cellular receptor 2, and granzyme B. Our findings were consistent in LUAD tissues, as corroborated using GEPIA. This highlights the fundamental role of HLA-DPA1 in the LUAD immune microenvironment.

Our study employed robust methodologies by combining data from TCGA and GEO databases for comprehensive bioinformatics analysis and conducting cell experiments to explore the role of HLA-DPA1 in LUAD progression. The incorporation of large datasets and cell experiments enhanced the reliability and validity of our findings. However, further research is needed to elucidate the mechanisms of HLA-DPA1 in LUAD progression and the underlying signaling pathways. Through our research, we revealed that HLA-DPA1 expression was significantly decreased in LUAD tissues and correlated with various clinical parameters. Furthermore, lower HLA-DPA1 expression was associated with reduced LUAD immune scores, while HLA-DPA1 overexpression was shown to inhibit cell proliferation and migration while promoting cell sensitivity to cisplatin.

## CONCLUSIONS

Decreased HLA-DPA1 expression is associated with poor prognosis, immune infiltration, cancer cell proliferation, and progression in LUAD. Our findings highlight the potential of HLA-DPA1 as both a prognostic biomarker and a therapeutic target for LUAD.

## MATERIALS AND METHODS

### Retrieval of data from TCGA and XENA databases

We downloaded expression data for 59 normal tissues and 535 samples of LUAD tissues from the TCGA (https://portal.gdc.cancer.gov/) database. Gene expression levels were measured in transcripts per million (TPM). Additionally, we downloaded data for 288 normal tissues from the GTEx database in XENA (https://xena.ucsc.edu/). Using TCGA database, we obtained clinical data from patients with LUAD, including cancer patient survival data updated up to January 2023.

### Retrieval of data from the GEO database

mRNA data from both LUAD and normal lung tissues were obtained from four relevant data sets, including GSE68571, GSE32863, GSE10072, and GSE2514 from the GEO (https://www.ncbi.nlm.nih.gov/geo) database. We retrieved series_matrix and platform annotation information from these datasets to verify the expression levels of HLA-DPA1 in LUAD based on gene annotation.

### Retrieval of data from the GEPIA database

To conduct further analyses, we utilized the GEPIA (http://gepia.cancer-pku.cn) database to analyze gene expression in relation to prognosis and other genes within LUAD [19]. We utilized the Pearson method within the correlation module of the GEPIA database to validate the relationship between HLA-DPA1 expression and immune cell markers in LUAD tissues.

### Retrieval of data from the UALCAN database

We utilized the UALCAN (http://ualcan.path.uab.edu) database to evaluate the HLA-DPA1 expression in both LUAD and normal tissues, focusing on its correlation with key clinical characteristics, such as cancer stage, tumor grade, race, and gender. Our study relied on data from the TCGA database, available within the UALCAN database [20].

### Retrieval of data from the TIMER database

The TIMER database is a widely employed tool in cancer research for investigating immune infiltrating cells [21]. We utilized the gene module within the TIMER (https://cistrome.shinyapps.io/timer/) database to analyze the relationship between HLA-DPA1 expression and immune infiltrating cells. Furthermore, we examined correlations between HLA-DPA1 expression and immune cell biomarkers specific to LUAD using the correlation analysis module.

### Diagnostic and prognostic values of HLA-DPA1

The diagnostic value of HLA-DPA1 in normal and LUAD tissues was assessed using receiver operating characteristic (ROC) analysis, with the area under the curve serving as the evaluation metric. Patient data on HLA-DPA1 expression were grouped by the median value, and survival analysis was employed to explore the relationship between decreased HLA-DPA1 expression and prognostic indicators in patients with LUAD. To evaluate the impact of decreased HLA-DPA1 expression on OS in patients with LUAD, we conducted a meta-analysis using the LCE (https://lce.biohpc.swmed.edu/) database.

### HLA-DPA1 co-expressed genes

In this study, we employed Pearson correlation analysis to identify genes strongly associated with HLA-DAP1 expression in LUAD tissues. A correlation was considered significant when the correlation coefficient (r) was greater than 0.6 or less than −0.6.

### Biological functions, and pathways of HLA-DPA1

Using the DAVID (https://david.ncifcrf.gov/) and TISIDB (http://cis.hku.hk/TISIDB/) databases, we performed GO and KEGG analyses to identify the pathways associated with the previously identified individual or multiple genes [22]. We used the DAVID database to identify significant functional annotations and mechanisms associated with genes that co-express with HLA-DPA1, applying a screening criterion based on *P*-values < 0.05. In addition to the DAVID analysis, we inputted the gene HLA-DPA1 into the TISIDB database to specifically investigate its functions and mechanisms.

### Cell models of HLA-DPA1 overexpression

A549 and A549/DDP cells were cultured in RPMI-1640 medium with 10% fetal bovine serum (FBS). The A549/DDP cells were cultured with 1 μg/mL cisplatin. HLA-DPA1 expression was induced by transfection using lipofectamine 3000 (Thermo Fisher Scientific, USA) according to the instructions of the manufacturer. The cells were cultured in optimal cell growth conditions in 6-well plates. The resulting expression was verified using reverse transcription-polymerase chain reaction (RT-PCR) and Western blotting. The NCBI identification number for HLA-DPA1 was NM_001242525.

### RT-PCR

Following cell transfection, RNA was extracted from A549 and A549/DDP cells using an RNA lysis solution. After quantification and normalization of RNA, reverse transcription was carried out to synthesize cDNA. The relative expression of HLA-DPA1 in both A549 and A549/DDP cells was calculated by subjecting the cDNA to PCR amplification using primers obtained from GeneCopoeia (China). The primer identification numbers provided for actin and HLA-DPA1 were HQP108762 and HQP008859, respectively.

### Western blotting

Total protein from A549 and A549/DDP cells was extracted using protein lysis solution following transfection. The BCA Kit (Solarbio, China) was employed to quantify and normalize protein levels. Western blotting was performed using standard methodologies for primary and secondary antibody incubations. The concentrations of tubulin (No. 11224-1-AP, Proteintech, China) and HLA-DPA1 (No. 16109-1-AP, Proteintech, China) were 1:5000 and 1:2000, respectively. The relative HLA-DPA1 expression levels in A549 and A549/DDP cells were determined after conducting the Western blotting.

### CCK-8 assay

Following the successful transfection of A549 and A549/DDP cells, viable cells were counted and plated at a density of 3,000 cells/well in a 96-well plate. Once the attachment was confirmed, cell activity was measured in both the control and HLA-DPA1 overexpression groups. The impact of cisplatin on cancer cell viability was assessed at concentrations of 0 μm, 4 μm, and 8 μm.

### Wound healing assay

After transfection, A549 and A549/DDP cells were counted and plated in 6-well plates. Straight lines were drawn on the surface of the plates using a 200 μl pipette tip. We included control and HLA-DPA1 overexpression groups. Photographs were taken after rinsing the wells with phosphate buffer solution (PBS) at 24 and 48 hours, respectively. Cell migration in the control and HLA-DPA1 overexpression groups was assessed, and results were compared for statistical significance.

### Transwell assay

The Matrigel was diluted and used to coat the bottom of the Transwell chamber. Suspensions of A549 and A549/DDP cells were prepared and added to the upper chamber of the Transwell chamber, while 600 μL of medium containing FBS was added to the lower chamber. After 24 hours of cultivation, the culture solution was discarded, and the cells were washed using PBS. Non-migrated cells in the upper chamber were gently removed using cotton swabs. A549 and A549/DDP cells were fixed using formaldehyde for 30 minutes and stained with 0.1% crystal violet staining solution for another 30 minutes. Subsequently, a cell count was performed.

### Relationship between HLA-DPA1 and the immune microenvironment

We evaluated the immune microenvironment in LUAD tissues by calculating immune scores and relative expression levels of immune cells using both single-sample Gene Set Enrichment Analysis and estimate methods. Pearson correlation analysis was used to investigate the relationship between HLA-DPA1 expression and the assessed immune characteristics. Additionally, we divided the samples into two groups based on the median value of HLA-DPA1 and examined the differences in immune scores and immune cell levels between these groups.

### Statistical analysis

We used the Wilcoxon rank sum test to determine the levels of HLA-DPA1 in LUAD tissues from TCGA, XENA, and GEO datasets. ROC, survival, and correlation analyses were employed to investigate the diagnostic value of HLA-DPA1 expression in LUAD, examine the relationship between HLA-DPA1 expression and patient prognosis, and analyze correlations between HLA-DPA1 expression and immune-related factors, respectively. Additionally, we used *t*-tests to evaluate the impact of HLA-DPA1 overexpression on cell proliferation, migration, and invasion. Data visualization and analysis were performed using Graph Prism software and the Xiantao Academic website (https://www.xiantaozi.com/products). For all statistical analyses, we considered a *P*-value of less than 0.05 statistically significant.

### Data availability

The data was available in the TCGA (https://portal.gdc.cancer.gov/), GEPIA (http://gepia.cancer-pku.cn), UALCAN (http://ualcan.path.uab.edu), XENA (https://xena.ucsc.edu/), Xiantao Academic (https://www.xiantaozi.com/products), TIMER (https://cistrome.shinyapps.io/timer/), DAVID (https://david.ncifcrf.gov/), LCE (https://lce.biohpc.swmed.edu/), TISIDB (http://cis.hku.hk/TISIDB/), and GEO (https://www.ncbi.nlm.nih.gov/geo) databases.

## Supplementary Materials

Supplementary Figures

Supplementary Tables 1 and 3

Supplementary Table 2
