# Supplementary material for: HLA-DPA1 overexpression inhibits cancer progression, reduces resistance to cisplatin, and correlates with increased immune infiltration in lung adenocarcinoma
Source: Aging (Albany NY). 2023 Oct 27;15(20):11067–91. doi: 10.18632/aging.205082 (PMC10637812; doi:10.18632/aging.205082)
Supplement: Supplementary Figures [file aging-15-205082-s001.pdf]

SUPPLEMENTARY FIGURES

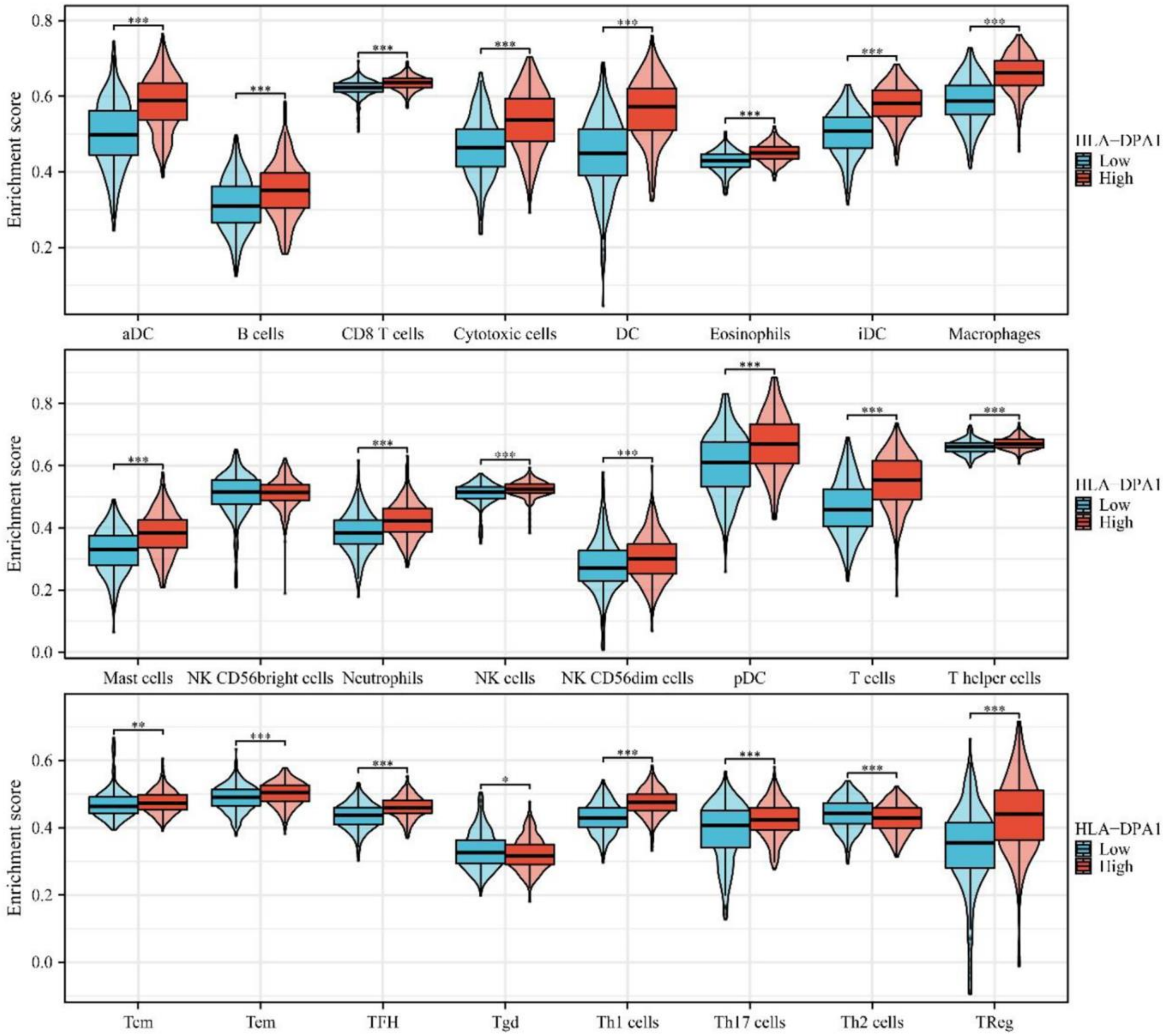

Supplementary Figure 1. The immune cells in high- and low-HLA-DPA1 expression groups.

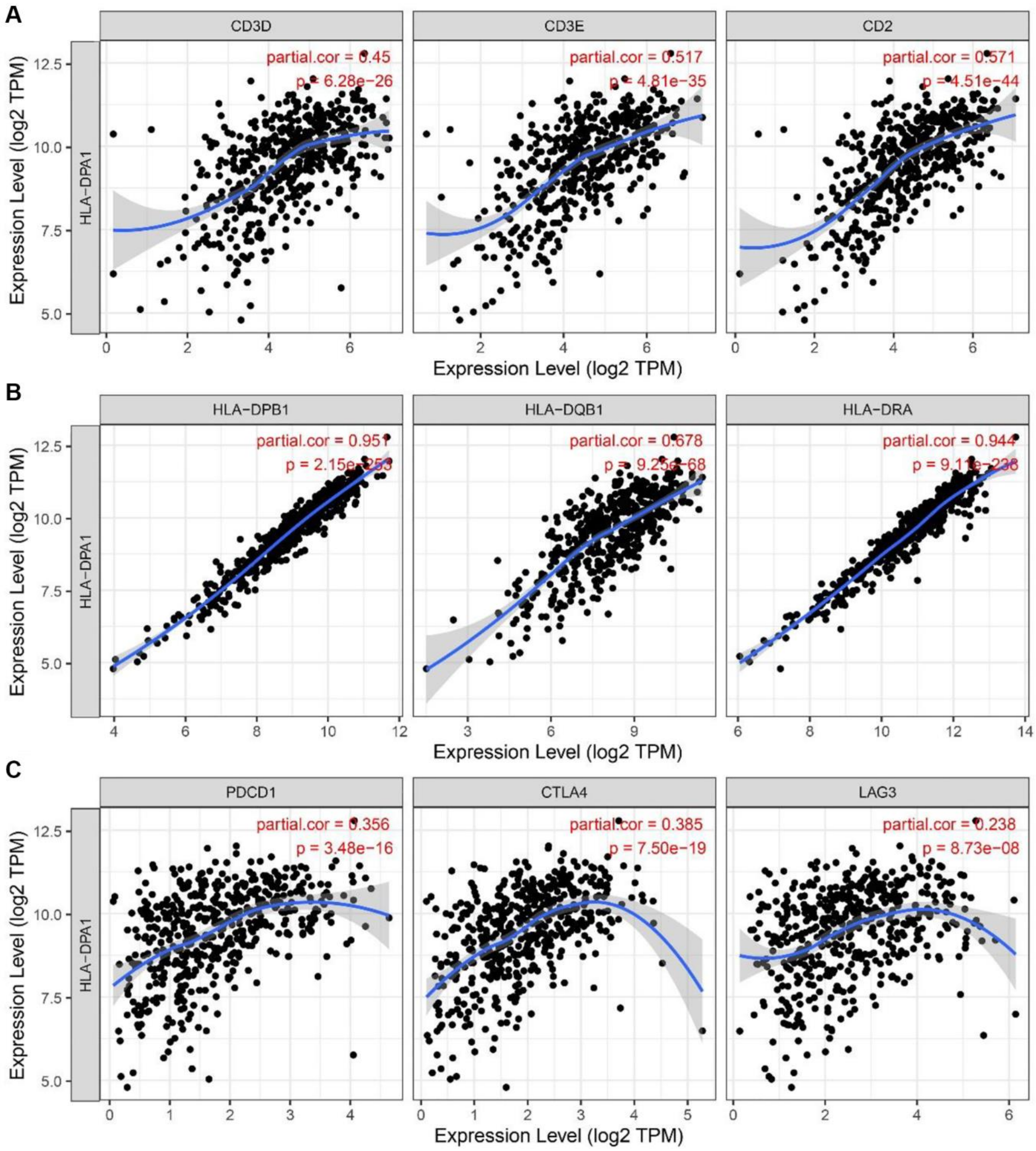

Supplementary Figure 2. The correlation between the HLA-DPA1 overexpression and immune cell markers in the context of tumor purity. (**A**) T cell markers; (**B**) Dendritic cell markers; (**C**) T cell exhaustion markers.
