# Supplementary material for: HLA-DPA1 overexpression inhibits cancer progression, reduces resistance to cisplatin, and correlates with increased immune infiltration in lung adenocarcinoma
Source: Aging (Albany NY). 2023 Oct 27;15(20):11067–91. doi: 10.18632/aging.205082 (PMC10637812; doi:10.18632/aging.205082)
Supplement: Supplementary Tables 1 and 3 [file aging-15-205082-s002.pdf]

SUPPLEMENTARY TABLES

Supplementary Table 1. Genes positively co-expressed with HLA-DPA1.

Gene	Coefficient	Gene	Coefficient	Gene	Coefficient	Gene	Coefficient
HLA-DPA1	1	GIMAP2	0.709154362	IL10RA	0.665266461	B2M	0.629901396
HLA-DRA	0.939929272	IRF8	0.707679199	FGR	0.663879355	HVCN1	0.628828632
HLA-DPB1	0.923988098	FCER1G	0.707343115	IL16	0.662454606	APBB1IP	0.628491844
CD74	0.899900908	LCP1	0.706873538	RAC2	0.661552796	APOBEC3G	0.628477428
HLA-DMB	0.89516136	WAS	0.706465797	CD40	0.656945302	PLD4	0.628209624
HLA-DRB1	0.894503912	FOLR2	0.706257982	STX11	0.656687429	GNGT2	0.627258125
HLA-DOA	0.884142667	HCK	0.705229462	ADORA3	0.656329789	SLC7A7	0.62652732
HLA-DQA1	0.865189432	TLR7	0.70445824	VSIG4	0.655937383	TRAC	0.626121154
HLA-DMA	0.847758139	EVI2B	0.70403413	AOAH	0.655612225	PLAAT4	0.625453196
HLA-DRB5	0.81288267	HLA-B	0.703761948	GPR65	0.655318311	CXorf21	0.625009856
CD4	0.797077423	JAML	0.702048268	CCR5	0.65510061	HLA-DRB9	0.624574107
HLA-DQB1	0.796030118	GMFG	0.70015895	CMKLR1	0.654985087	CSF2RA	0.624137366
ITGB2	0.788547922	MPEG1	0.698885411	SIGLEC7	0.65427375	PIK3R6	0.623917277
LY86	0.785160735	SIGLEC9	0.698402631	ARHGDIB	0.653929457	PTPRO	0.623299519
MNDA	0.783246088	NAPSB	0.69803603	NCF2	0.652792325	SLC37A2	0.622955534
AIF1	0.772757553	C1QC	0.695624592	ALOX5AP	0.652546315	PTGER4	0.621546807
TNFAIP8L2	0.766436301	CD300C	0.695043028	APOL3	0.652251108	TRAV9-2	0.621295947
BTK	0.764783007	TREM2	0.694295369	CSF2RB	0.651149346	HPGDS	0.621078907
SASH3	0.759361863	ITGAM	0.693786636	BIN2	0.650272112	LAT2	0.619846708
RNASE6	0.758584752	LCP2	0.693319357	CCR2	0.649746926	PTAFR	0.619758083
LST1	0.757025543	C1QB	0.693105046	FCGR2A	0.649034746	GIMAP7	0.619382155
SNX20	0.756369058	ABI3	0.69262654	TESPA1	0.648898786	GIMAP1	0.618293486
SELPLG	0.75520627	CD83	0.69206892	CPVL	0.648893369	DPYD	0.617323461
CASP1	0.754163424	P2RX7	0.691813207	MSR1	0.648375003	LIPA	0.61652342
HLA-E	0.753729746	CTSS	0.691611696	TRBV20-1	0.647268782	SUSD3	0.61648575
LAPTM5	0.753479911	GIMAP4	0.688455211	SIGLEC1	0.645604697	GAPT	0.61581821
MS4A6A	0.752081068	P2RY12	0.687804712	STAT5A	0.645207313	CTSO	0.615547705
NCKAP1L	0.751531026	ARHGEF6	0.684457645	GIMAP6	0.644978996	GPR34	0.615511763
NCF4	0.750258443	C1QA	0.683927379	PIK3AP1	0.64456783	SLC9A9	0.612763332
CSF1R	0.749004745	FPR3	0.683479884	TRPV2	0.643274177	PTPN7	0.612669918
CD33	0.745531105	PILRA	0.683236633	RASGRP4	0.642443942	GAB3	0.611165095
LAIR1	0.745137597	TRIM22	0.682640635	PPM1M	0.64024745	SIRPA	0.610996844
DOK2	0.743745445	PLEKHO2	0.68236022	CD2	0.639726955	BTN2A2	0.610550918
IGSF6	0.742384755	TNFSF13B	0.682305822	MS4A7	0.639638351	CD14	0.610491259
HAVCR2	0.741026835	FERMT3	0.681442247	VAV1	0.639063669	TNFRSF1B	0.609933332
SPN	0.740607311	CARD16	0.680719773	ARHGAP25	0.638706387	SOWAHD	0.60992759
PLEK	0.739850317	MYO1F	0.680453501	PIK3R5	0.638646893	NLRP3	0.609720717
C1orf162	0.736860456	MS4A4A	0.679303416	FGL2	0.637921546	SIRPB2	0.609692421
CD53	0.734130409	ARHGAP30	0.678961328	IL12RB1	0.637371434	GLIPR2	0.608938195
SPI1	0.733722089	CYTH4	0.678864793	CD48	0.637292106	LYZ	0.607803012
CIITA	0.733256993	SLAMF8	0.67822393	DPEP2	0.636755608	PLXNC1	0.606206182
CLEC10A	0.7311992	TMEM273	0.676954861	FCGR3A	0.636637542	RGS18	0.605792372
NFAM1	0.728820941	LRRC25	0.676003165	IKZF1	0.636397279	ARHGAP15	0.605765121

P2RY13	0.728515817	DOCK2	0.675190718	RTN1	0.635615729	PIK3CG	0.605572854
LPXN	0.728140858	FGD2	0.675172682	C16orf54	0.634502003	ACP5	0.605251514
HLA-DQB2	0.726972607	TLR2	0.674083278	FAM78A	0.634174009	RCSD1	0.604741018
CD300LF	0.726079309	BTN3A3	0.673399207	OLR1	0.633164644	APOBEC3C	0.604032018
C3AR1	0.726078036	TLR8	0.672881567	OSCAR	0.632783049	CD1C	0.603010184
CD86	0.725319008	PARVG	0.671433867	DOCK8	0.631741926	ARHGAP9	0.602774842
GGTA1	0.723482082	PTPRC	0.670614401	FCGR1A	0.631655081	GPNMB	0.602568483
CYBB	0.722112758	TAGAP	0.670340181	SLA	0.631621015	HCST	0.602329332
SLC15A3	0.720823718	LSP1	0.670221883	CRTAM	0.631082981	TRBV19	0.601083604
CD37	0.718415678	ALOX5	0.669901347	HLA-F	0.630999906	NRROS	0.600937344
CD52	0.71295562	ITGAL	0.668225928	LILRB1	0.630278903	NCF1	0.600869284
SCIMP	0.711263816	CD84	0.6679874	MRC1	0.630169002	PSTPIP1	0.6008261
SLCO2B1	0.710385258	HLA-DOB	0.667899962	CLEC7A	0.630093914	LGALS9	0.600292892
TYROBP	0.709641209	SAMHD1	0.666470219	PTPN22	0.630090261	TRAV8-2	0.600056566
CLECL1	0.6000455						

Supplementary Table 3. The biological functions of HLA-DPA1 using GO analysis in the TISIDB database.

GO type	Term
BP	GO:0001819 positive regulation of cytokine production
BP	GO:0002429 immune response-activating cell surface receptor signaling pathway
BP	GO:0002478 antigen processing and presentation of exogenous peptide antigen
BP	GO:0002495 antigen processing and presentation of peptide antigen via MHC class II
BP	GO:0002504 antigen processing and presentation of peptide or polysaccharide antigen via MHC class II
BP	GO:0002694 regulation of leukocyte activation
BP	GO:0002696 positive regulation of leukocyte activation
BP	GO:0002757 immune response-activating signal transduction
BP	GO:0002764 immune response-regulating signaling pathway
BP	GO:0002768 immune response-regulating cell surface receptor signaling pathway
BP	GO:0007159 leukocyte cell-cell adhesion
BP	GO:0019882 antigen processing and presentation
BP	GO:0019884 antigen processing and presentation of exogenous antigen
BP	GO:0019886 antigen processing and presentation of exogenous peptide antigen via MHC class II
BP	GO:0022407 regulation of cell-cell adhesion
BP	GO:0022409 positive regulation of cell-cell adhesion
BP	GO:0031294 lymphocyte costimulation
BP	GO:0031295 T cell costimulation
BP	GO:0032609 interferon-gamma production
BP	GO:0032649 regulation of interferon-gamma production
BP	GO:0032729 positive regulation of interferon-gamma production
BP	GO:0032943 mononuclear cell proliferation
BP	GO:0032944 regulation of mononuclear cell proliferation
BP	GO:0032946 positive regulation of mononuclear cell proliferation
BP	GO:0034341 response to interferon-gamma
BP	GO:0042098 T cell proliferation
BP	GO:0042102 positive regulation of T cell proliferation
BP	GO:0042110 T cell activation

BP	GO:0042129 regulation of T cell proliferation
BP	GO:0045785 positive regulation of cell adhesion
BP	GO:0046651 lymphocyte proliferation
BP	GO:0048002 antigen processing and presentation of peptide antigen
BP	GO:0050670 regulation of lymphocyte proliferation
BP	GO:0050671 positive regulation of lymphocyte proliferation
BP	GO:0050851 antigen receptor-mediated signaling pathway
BP	GO:0050852 T cell receptor signaling pathway
BP	GO:0050863 regulation of T cell activation
BP	GO:0050865 regulation of cell activation
BP	GO:0050867 positive regulation of cell activation
BP	GO:0050870 positive regulation of T cell activation
BP	GO:0051249 regulation of lymphocyte activation
BP	GO:0051251 positive regulation of lymphocyte activation
BP	GO:0060333 interferon-gamma-mediated signaling pathway
BP	GO:0070486 leukocyte aggregation
BP	GO:0070489 T cell aggregation
BP	GO:0070661 leukocyte proliferation
BP	GO:0070663 regulation of leukocyte proliferation
BP	GO:0070665 positive regulation of leukocyte proliferation
BP	GO:0071346 cellular response to interferon-gamma
BP	GO:0071593 lymphocyte aggregation
BP	GO:1903037 regulation of leukocyte cell-cell adhesion
BP	GO:1903039 positive regulation of leukocyte cell-cell adhesion
MF	GO:0003823 antigen binding
MF	GO:0032395 MHC class II receptor activity
MF	GO:0033218 amide binding
MF	GO:0042277 peptide binding
MF	GO:0042605 peptide antigen binding
CC	GO:0005765 lysosomal membrane
CC	GO:0005802 trans-Golgi network
CC	GO:0010008 endosome membrane
CC	GO:0012507 ER to Golgi transport vesicle membrane
CC	GO:0030133 transport vesicle
CC	GO:0030134 ER to Golgi transport vesicle
CC	GO:0030135 coated vesicle
CC	GO:0030136 clathrin-coated vesicle
CC	GO:0030139 endocytic vesicle
CC	GO:0030176 integral component of endoplasmic reticulum membrane
CC	GO:0030658 transport vesicle membrane
CC	GO:0030659 cytoplasmic vesicle membrane
CC	GO:0030662 coated vesicle membrane
CC	GO:0030665 clathrin-coated vesicle membrane
CC	GO:0030666 endocytic vesicle membrane
CC	GO:0030669 clathrin-coated endocytic vesicle membrane
CC	GO:0031227 intrinsic component of endoplasmic reticulum membrane
CC	GO:0031984 organelle subcompartment

CC	GO:0032588 trans-Golgi network membrane
CC	GO:0042611 MHC protein complex
CC	GO:0042613 MHC class II protein complex
CC	GO:0044440 endosomal part
CC	GO:0045334 clathrin-coated endocytic vesicle
CC	GO:0071556 integral component of lumenal side of endoplasmic reticulum membrane
CC	GO:0098552 side of membrane
CC	GO:0098553 lumenal side of endoplasmic reticulum membrane
CC	GO:0098791 Golgi subcompartment
CC	GO:0098852 lytic vacuole membrane

Abbreviations: GO: gene ontology; BP: biological process; MF: molecular function; CC: cellular component.
