# Supplementary material for: HLA-DPA1 overexpression inhibits cancer progression, reduces resistance to cisplatin, and correlates with increased immune infiltration in lung adenocarcinoma
Source: Aging (Albany NY). 2023 Oct 27;15(20):11067–91. doi: 10.18632/aging.205082 (PMC10637812; doi:10.18632/aging.205082)
Supplement: Supplementary Table 2 [file aging-15-205082-s003.docx]

**Supplementary Table 2. The biological functions of genes co-expressed with HLA-DPA1 using GO analysis in the DAVID database.**

| GO type | Term | Count | P |
| --- | --- | --- | --- |
| BP | GO:0006955~immune response | 49 | 8.79E-32 |
| CC | GO:0005886~plasma membrane | 139 | 5.51E-30 |
| CC | GO:0042613~MHC class II protein complex | 16 | 1.42E-23 |
| BP | GO:0006954~inflammatory response | 38 | 2.61E-23 |
| BP | GO:0002381~immunoglobulin production involved in immunoglobulin mediated immune response | 14 | 4.82E-23 |
| BP | GO:0019886~antigen processing and presentation of exogenous peptide antigen via MHC class II | 16 | 1.05E-21 |
| BP | GO:0050870~positive regulation of T cell activation | 16 | 3.37E-21 |
| CC | GO:0009897~external side of plasma membrane | 38 | 4.02E-21 |
| MF | GO:0023026~MHC class II protein complex binding | 15 | 8.45E-21 |
| BP | GO:0002503~peptide antigen assembly with MHC class II protein complex | 13 | 1.21E-20 |
| BP | GO:0002504~antigen processing and presentation of peptide or polysaccharide antigen via MHC class II | 13 | 2.38E-19 |
| BP | GO:0019882~antigen processing and presentation | 15 | 2.63E-17 |
| CC | GO:0016021~integral component of membrane | 118 | 3.29E-17 |
| CC | GO:0009986~cell surface | 38 | 4.69E-17 |
| CC | GO:0005887~integral component of plasma membrane | 56 | 6.14E-17 |
| BP | GO:0045087~innate immune response | 37 | 1.21E-16 |
| BP | GO:0002250~adaptive immune response | 32 | 1.96E-16 |
| MF | GO:0032395~MHC class II receptor activity | 10 | 1.36E-15 |
| CC | GO:0098553~lumenal side of endoplasmic reticulum membrane | 12 | 5.51E-15 |
| CC | GO:0071556~integral component of lumenal side of endoplasmic reticulum membrane | 12 | 5.51E-15 |
| BP | GO:0032729~positive regulation of interferon-gamma production | 16 | 6.40E-15 |
| BP | GO:0032760~positive regulation of tumor necrosis factor production | 17 | 3.49E-14 |
| CC | GO:0012507~ER to Golgi transport vesicle membrane | 13 | 8.93E-13 |
| CC | GO:0010008~endosome membrane | 20 | 5.97E-11 |
| BP | GO:0001916~positive regulation of T cell mediated cytotoxicity | 10 | 1.34E-10 |
| MF | GO:0042605~peptide antigen binding | 10 | 3.15E-10 |
| MF | GO:0038023~signaling receptor activity | 18 | 4.81E-10 |
| BP | GO:0032755~positive regulation of interleukin-6 production | 13 | 1.32E-09 |
| CC | GO:0016020~membrane | 79 | 1.43E-09 |
| BP | GO:0042102~positive regulation of T cell proliferation | 11 | 2.76E-09 |
| BP | GO:0007166~cell surface receptor signaling pathway | 20 | 2.83E-09 |
| CC | GO:0030669~clathrin-coated endocytic vesicle membrane | 11 | 4.09E-09 |
| BP | GO:0007165~signal transduction | 39 | 9.14E-09 |
| BP | GO:0006935~chemotaxis | 13 | 1.02E-08 |
| CC | GO:0030670~phagocytic vesicle membrane | 11 | 1.06E-08 |
| CC | GO:0030658~transport vesicle membrane | 9 | 1.44E-08 |
| CC | GO:0035579~specific granule membrane | 11 | 5.43E-08 |
| CC | GO:0030666~endocytic vesicle membrane | 10 | 7.27E-08 |
| CC | GO:0070821~tertiary granule membrane | 10 | 9.30E-08 |
| BP | GO:0006968~cellular defense response | 9 | 1.02E-07 |
| BP | GO:0071346~cellular response to interferon-gamma | 11 | 1.05E-07 |
| BP | GO:0042110~T cell activation | 9 | 1.18E-07 |
| MF | GO:0005102~receptor binding | 20 | 1.35E-07 |
| MF | GO:0004888~transmembrane signaling receptor activity | 14 | 1.58E-07 |
| BP | GO:0060333~interferon-gamma-mediated signaling pathway | 7 | 3.82E-07 |
| BP | GO:0032753~positive regulation of interleukin-4 production | 7 | 3.82E-07 |
| BP | GO:0032735~positive regulation of interleukin-12 production | 8 | 4.51E-07 |
| BP | GO:0045730~respiratory burst | 6 | 8.16E-07 |
| BP | GO:0071222~cellular response to lipopolysaccharide | 13 | 9.20E-07 |
| BP | GO:0002480~antigen processing and presentation of exogenous peptide antigen via MHC class I, TAP-independent | 5 | 9.28E-07 |
| BP | GO:0050830~defense response to Gram-positive bacterium | 11 | 1.63E-06 |
| CC | GO:0030667~secretory granule membrane | 10 | 1.67E-06 |
| CC | GO:0032588~trans-Golgi network membrane | 10 | 1.97E-06 |
| BP | GO:0032720~negative regulation of tumor necrosis factor production | 9 | 2.13E-06 |
| BP | GO:0032743~positive regulation of interleukin-2 production | 7 | 2.32E-06 |
| BP | GO:0006952~defense response | 9 | 2.84E-06 |
| CC | GO:0005765~lysosomal membrane | 17 | 4.07E-06 |
| BP | GO:0031663~lipopolysaccharide-mediated signaling pathway | 7 | 5.91E-06 |
| BP | GO:0032731~positive regulation of interleukin-1 beta production | 8 | 6.07E-06 |
| BP | GO:0045321~leukocyte activation | 5 | 6.34E-06 |
| BP | GO:0032757~positive regulation of interleukin-8 production | 8 | 6.74E-06 |
| CC | GO:0001772~immunological synapse | 7 | 8.64E-06 |
| BP | GO:1901224~positive regulation of NIK/NF-kappaB signaling | 8 | 9.13E-06 |
| BP | GO:0051607~defense response to virus | 13 | 9.60E-06 |
| BP | GO:0030890~positive regulation of B cell proliferation | 7 | 1.02E-05 |
| BP | GO:0001774~microglial cell activation | 6 | 1.18E-05 |
| BP | GO:0034154~toll-like receptor 7 signaling pathway | 4 | 1.26E-05 |
| BP | GO:0042130~negative regulation of T cell proliferation | 7 | 1.48E-05 |
| BP | GO:0050766~positive regulation of phagocytosis | 7 | 1.66E-05 |
| BP | GO:0050852~T cell receptor signaling pathway | 10 | 1.86E-05 |
| CC | GO:0044853~plasma membrane raft | 6 | 2.26E-05 |
| CC | GO:0043020~NADPH oxidase complex | 5 | 3.64E-05 |
| BP | GO:0070374~positive regulation of ERK1 and ERK2 cascade | 12 | 3.96E-05 |
| BP | GO:0006911~phagocytosis, engulfment | 9 | 4.05E-05 |
| CC | GO:0000139~Golgi membrane | 21 | 4.34E-05 |
| CC | GO:0031901~early endosome membrane | 11 | 4.82E-05 |
| BP | GO:0051056~regulation of small GTPase mediated signal transduction | 9 | 4.86E-05 |
| BP | GO:0019221~cytokine-mediated signaling pathway | 10 | 5.48E-05 |
| BP | GO:0032930~positive regulation of superoxide anion generation | 5 | 5.80E-05 |
| BP | GO:0050776~regulation of immune response | 6 | 6.25E-05 |
| BP | GO:0038094~Fc-gamma receptor signaling pathway | 4 | 6.87E-05 |
| BP | GO:0032733~positive regulation of interleukin-10 production | 6 | 8.99E-05 |
| MF | GO:0042802~identical protein binding | 37 | 9.67E-05 |
| CC | GO:0042612~MHC class I protein complex | 4 | 1.00E-04 |
| BP | GO:0006909~phagocytosis | 7 | 1.15E-04 |
| BP | GO:0042554~superoxide anion generation | 5 | 1.23E-04 |
| BP | GO:0032722~positive regulation of chemokine production | 6 | 1.26E-04 |
| BP | GO:0060907~positive regulation of macrophage cytokine production | 5 | 1.45E-04 |
| BP | GO:0032496~response to lipopolysaccharide | 9 | 1.68E-04 |
| BP | GO:0002639~positive regulation of immunoglobulin production | 5 | 1.98E-04 |
| MF | GO:0019864~IgG binding | 4 | 2.03E-04 |
| MF | GO:0042608~T cell receptor binding | 4 | 2.03E-04 |
| BP | GO:0007229~integrin-mediated signaling pathway | 8 | 2.16E-04 |
| BP | GO:0008360~regulation of cell shape | 9 | 2.44E-04 |
| BP | GO:0030225~macrophage differentiation | 5 | 3.01E-04 |
| CC | GO:0045121~membrane raft | 11 | 3.12E-04 |
| BP | GO:0043123~positive regulation of I-kappaB kinase/NF-kappaB signaling | 10 | 3.33E-04 |
| BP | GO:0045953~negative regulation of natural killer cell mediated cytotoxicity | 4 | 3.37E-04 |
| BP | GO:0035589~G-protein coupled purinergic nucleotide receptor signaling pathway | 4 | 3.37E-04 |
| MF | GO:0045028~G-protein coupled purinergic nucleotide receptor activity | 4 | 3.46E-04 |
| CC | GO:0005602~complement component C1 complex | 3 | 3.48E-04 |
| MF | GO:0001540~beta-amyloid binding | 7 | 3.50E-04 |
| BP | GO:0002519~natural killer cell tolerance induction | 3 | 3.52E-04 |
| BP | GO:0007159~leukocyte cell-cell adhesion | 5 | 3.88E-04 |
| CC | GO:0005884~actin filament | 7 | 4.15E-04 |
| BP | GO:0045580~regulation of T cell differentiation | 4 | 4.25E-04 |
| BP | GO:0002376~immune system process | 6 | 4.56E-04 |
| BP | GO:0019722~calcium-mediated signaling | 7 | 5.11E-04 |
| BP | GO:0046598~positive regulation of viral entry into host cell | 4 | 5.27E-04 |
| BP | GO:0006959~humoral immune response | 6 | 5.32E-04 |
| CC | GO:0005764~lysosome | 12 | 5.98E-04 |
| BP | GO:0007169~transmembrane receptor protein tyrosine kinase signaling pathway | 8 | 6.46E-04 |
| CC | GO:0005944~phosphatidylinositol 3-kinase complex, class IB | 3 | 6.91E-04 |
| BP | GO:0002291~T cell activation via T cell receptor contact with antigen bound to MHC molecule on antigen presenting cell | 3 | 6.99E-04 |
| BP | GO:1904151~positive regulation of microglial cell mediated cytotoxicity | 3 | 6.99E-04 |
| BP | GO:0035696~monocyte extravasation | 3 | 6.99E-04 |
| BP | GO:0071226~cellular response to molecule of fungal origin | 3 | 6.99E-04 |
| BP | GO:0043315~positive regulation of neutrophil degranulation | 3 | 6.99E-04 |
| MF | GO:0001530~lipopolysaccharide binding | 5 | 7.83E-04 |
| BP | GO:0050868~negative regulation of T cell activation | 4 | 9.23E-04 |
| BP | GO:0050727~regulation of inflammatory response | 7 | 9.29E-04 |
| MF | GO:0038187~pattern recognition receptor activity | 4 | 9.47E-04 |
| CC | GO:0045335~phagocytic vesicle | 6 | 9.74E-04 |
| BP | GO:0032695~negative regulation of interleukin-12 production | 4 | 0.001087685 |
| BP | GO:0006801~superoxide metabolic process | 4 | 0.001087685 |
| BP | GO:0042098~T cell proliferation | 5 | 0.001110391 |
| CC | GO:0032010~phagolysosome | 3 | 0.001143253 |
| BP | GO:0007155~cell adhesion | 16 | 0.001149767 |
| BP | GO:0050729~positive regulation of inflammatory response | 7 | 0.001188139 |
| CC | GO:0055038~recycling endosome membrane | 7 | 0.001210014 |
| BP | GO:0032691~negative regulation of interleukin-1 beta production | 5 | 0.001324047 |
| BP | GO:0032689~negative regulation of interferon-gamma production | 5 | 0.001440953 |
| BP | GO:0007204~positive regulation of cytosolic calcium ion concentration | 8 | 0.001465181 |
| BP | GO:0016064~immunoglobulin mediated immune response | 4 | 0.001469132 |
| BP | GO:0045088~regulation of innate immune response | 4 | 0.001469132 |
| BP | GO:0043406~positive regulation of MAP kinase activity | 6 | 0.001534827 |
| BP | GO:0006898~receptor-mediated endocytosis | 6 | 0.001625646 |
| BP | GO:0043372~positive regulation of CD4-positive, alpha-beta T cell differentiation | 3 | 0.001723475 |
| MF | GO:0001875~lipopolysaccharide receptor activity | 3 | 0.001753809 |
| MF | GO:0038024~cargo receptor activity | 4 | 0.001973978 |
| BP | GO:0030593~neutrophil chemotaxis | 6 | 0.002028774 |
| BP | GO:0051092~positive regulation of NF-kappaB transcription factor activity | 8 | 0.002251793 |
| CC | GO:1904813~ficolin-1-rich granule lumen | 7 | 0.002334468 |
| BP | GO:0090023~positive regulation of neutrophil chemotaxis | 4 | 0.002460472 |
| BP | GO:2000249~regulation of actin cytoskeleton reorganization | 4 | 0.002460472 |
| MF | GO:1901981~phosphatidylinositol phosphate binding | 4 | 0.002522527 |
| BP | GO:0002224~toll-like receptor signaling pathway | 4 | 0.002759237 |
| BP | GO:0001782~B cell homeostasis | 4 | 0.002759237 |
| CC | GO:0070062~extracellular exosome | 39 | 0.002929456 |
| MF | GO:0003953~NAD+ nucleosidase activity | 4 | 0.003156643 |
| BP | GO:0098883~synapse disassembly | 3 | 0.00317118 |
| MF | GO:0042609~CD4 receptor binding | 3 | 0.003226586 |
| CC | GO:0001891~phagocytic cup | 4 | 0.003363802 |
| BP | GO:0050728~negative regulation of inflammatory response | 7 | 0.003404079 |
| BP | GO:0032703~negative regulation of interleukin-2 production | 4 | 0.003421488 |
| BP | GO:0050829~defense response to Gram-negative bacterium | 6 | 0.003675083 |
| BP | GO:0034113~heterotypic cell-cell adhesion | 4 | 0.003785909 |
| MF | GO:0004896~cytokine receptor activity | 5 | 0.003792296 |
| CC | GO:0005943~phosphatidylinositol 3-kinase complex, class IA | 3 | 0.003999589 |
| BP | GO:0043382~positive regulation of memory T cell differentiation | 3 | 0.004048034 |
| BP | GO:0048873~homeostasis of number of cells within a tissue | 4 | 0.004173113 |
| CC | GO:0005576~extracellular region | 37 | 0.004330759 |
| CC | GO:0002102~podosome | 4 | 0.004506827 |
| MF | GO:0005085~guanyl-nucleotide exchange factor activity | 9 | 0.004938089 |
| CC | GO:0072559~NLRP3 inflammasome complex | 3 | 0.004963921 |
| CC | GO:0030027~lamellipodium | 8 | 0.005011322 |
| BP | GO:0002862~negative regulation of inflammatory response to antigenic stimulus | 4 | 0.005017486 |
| BP | GO:0045657~positive regulation of monocyte differentiation | 3 | 0.005023838 |
| BP | GO:0050764~regulation of phagocytosis | 3 | 0.005023838 |
| BP | GO:0031666~positive regulation of lipopolysaccharide-mediated signaling pathway | 3 | 0.005023838 |
| BP | GO:0050863~regulation of T cell activation | 3 | 0.005023838 |
| BP | GO:0002474~antigen processing and presentation of peptide antigen via MHC class I | 3 | 0.005023838 |
| MF | GO:0016175~superoxide-generating NADPH oxidase activity | 3 | 0.005110969 |
| MF | GO:0005096~GTPase activator activity | 10 | 0.00512803 |
| BP | GO:0001819~positive regulation of cytokine production | 5 | 0.005837367 |
| BP | GO:0002230~positive regulation of defense response to virus by host | 4 | 0.005957637 |
| BP | GO:0001818~negative regulation of cytokine production | 4 | 0.005957637 |
| BP | GO:0032675~regulation of interleukin-6 production | 3 | 0.006096352 |
| BP | GO:0071223~cellular response to lipoteichoic acid | 3 | 0.006096352 |
| BP | GO:0045060~negative thymic T cell selection | 3 | 0.006096352 |
| BP | GO:0045059~positive thymic T cell selection | 3 | 0.006096352 |
| BP | GO:1900015~regulation of cytokine production involved in inflammatory response | 3 | 0.006096352 |
| MF | GO:0016176~superoxide-generating NADPH oxidase activator activity | 3 | 0.006201695 |
| MF | GO:0004875~complement receptor activity | 3 | 0.006201695 |
| MF | GO:0001851~complement component C3b binding | 3 | 0.006201695 |
| MF | GO:0071723~lipopeptide binding | 3 | 0.006201695 |
| BP | GO:0007160~cell-matrix adhesion | 6 | 0.006864292 |
| BP | GO:0042113~B cell activation | 4 | 0.006996263 |
| CC | GO:0005925~focal adhesion | 12 | 0.007012042 |
| MF | GO:0030246~carbohydrate binding | 8 | 0.007117197 |
| CC | GO:0001931~uropod | 3 | 0.00717734 |
| BP | GO:0033674~positive regulation of kinase activity | 5 | 0.007545729 |
| BP | GO:0048678~response to axon injury | 4 | 0.007553261 |
| BP | GO:0032715~negative regulation of interleukin-6 production | 5 | 0.007922795 |
| MF | GO:0005178~integrin binding | 7 | 0.008123724 |
| BP | GO:1901216~positive regulation of neuron death | 4 | 0.008135748 |
| BP | GO:0032819~positive regulation of natural killer cell proliferation | 3 | 0.008522732 |
| BP | GO:0002430~complement receptor mediated signaling pathway | 3 | 0.008522732 |
| BP | GO:0002357~defense response to tumor cell | 3 | 0.008522732 |
| BP | GO:0097242~beta-amyloid clearance | 3 | 0.008522732 |
| CC | GO:0031226~intrinsic component of plasma membrane | 4 | 0.008600704 |
| BP | GO:0001934~positive regulation of protein phosphorylation | 8 | 0.008758519 |
| MF | GO:0005515~protein binding | 156 | 0.00909291 |
| CC | GO:0043231~intracellular membrane-bounded organelle | 20 | 0.009135382 |
| BP | GO:0071260~cellular response to mechanical stimulus | 5 | 0.009554287 |
| BP | GO:0097190~apoptotic signaling pathway | 5 | 0.009554287 |
| BP | GO:0006691~leukotriene metabolic process | 3 | 0.009872297 |
| BP | GO:0045579~positive regulation of B cell differentiation | 3 | 0.009872297 |
| BP | GO:0032728~positive regulation of interferon-beta production | 4 | 0.010038571 |
| CC | GO:0043235~receptor complex | 8 | 0.01007271 |
| MF | GO:0001618~virus receptor activity | 5 | 0.010296053 |
| BP | GO:0007264~small GTPase mediated signal transduction | 6 | 0.010525494 |
| BP | GO:0030217~T cell differentiation | 4 | 0.010725354 |
| BP | GO:0030316~osteoclast differentiation | 4 | 0.010725354 |
| BP | GO:0032736~positive regulation of interleukin-13 production | 3 | 0.011309971 |
| BP | GO:0071639~positive regulation of monocyte chemotactic protein-1 production | 3 | 0.011309971 |
| BP | GO:0097028~dendritic cell differentiation | 3 | 0.011309971 |
| BP | GO:0007259~JAK-STAT cascade | 4 | 0.01143871 |
| BP | GO:0046007~negative regulation of activated T cell proliferation | 3 | 0.012833692 |
| BP | GO:0034142~toll-like receptor 4 signaling pathway | 3 | 0.012833692 |
| BP | GO:0002486~antigen processing and presentation of endogenous peptide antigen via MHC class I via ER pathway, TAP-independent | 3 | 0.012833692 |
| BP | GO:0042100~B cell proliferation | 4 | 0.012945797 |
| BP | GO:0014065~phosphatidylinositol 3-kinase signaling | 4 | 0.012945797 |
| MF | GO:0019955~cytokine binding | 4 | 0.013254097 |
| BP | GO:0014068~positive regulation of phosphatidylinositol 3-kinase signaling | 5 | 0.013984747 |
| BP | GO:0002407~dendritic cell chemotaxis | 3 | 0.014441431 |
| BP | GO:0002476~antigen processing and presentation of endogenous peptide antigen via MHC class Ib | 3 | 0.014441431 |
| BP | GO:0070527~platelet aggregation | 4 | 0.014560984 |
| CC | GO:0031902~late endosome membrane | 6 | 0.015436545 |
| MF | GO:0001784~phosphotyrosine binding | 4 | 0.015773176 |
| BP | GO:0042742~defense response to bacterium | 8 | 0.016072599 |
| MF | GO:0042834~peptidoglycan binding | 3 | 0.016402767 |
| BP | GO:0050853~B cell receptor signaling pathway | 6 | 0.016740752 |
| BP | GO:0043087~regulation of GTPase activity | 5 | 0.017558274 |
| BP | GO:0050731~positive regulation of peptidyl-tyrosine phosphorylation | 5 | 0.017558274 |
| BP | GO:0050921~positive regulation of chemotaxis | 3 | 0.017901018 |
| BP | GO:0010759~positive regulation of macrophage chemotaxis | 3 | 0.017901018 |
| BP | GO:0030889~negative regulation of B cell proliferation | 3 | 0.017901018 |
| BP | GO:2000406~positive regulation of T cell migration | 3 | 0.017901018 |
| BP | GO:0031668~cellular response to extracellular stimulus | 3 | 0.017901018 |
| CC | GO:0005911~cell-cell junction | 7 | 0.017995978 |
| BP | GO:0007162~negative regulation of cell adhesion | 4 | 0.018119107 |
| MF | GO:0004950~chemokine receptor activity | 3 | 0.01820126 |
| BP | GO:0018108~peptidyl-tyrosine phosphorylation | 6 | 0.018223462 |
| BP | GO:0000902~cell morphogenesis | 5 | 0.018864487 |
| BP | GO:0032088~negative regulation of NF-kappaB transcription factor activity | 5 | 0.018864487 |
| BP | GO:0098609~cell-cell adhesion | 7 | 0.019331716 |
| MF | GO:1990782~protein tyrosine kinase binding | 4 | 0.019522448 |
| BP | GO:0050850~positive regulation of calcium-mediated signaling | 3 | 0.019748975 |
| MF | GO:0042288~MHC class I protein binding | 3 | 0.02007897 |
| CC | GO:0097179~protease inhibitor complex | 2 | 0.021547344 |
| CC | GO:0034688~integrin alphaM-beta2 complex | 2 | 0.021547344 |
| CC | GO:0036398~TCR signalosome | 2 | 0.021547344 |
| CC | GO:0032398~MHC class Ib protein complex | 2 | 0.021547344 |
| CC | GO:0034687~integrin alphaL-beta2 complex | 2 | 0.021547344 |
| BP | GO:0038096~Fc-gamma receptor signaling pathway involved in phagocytosis | 3 | 0.021673169 |
| BP | GO:2000473~positive regulation of hematopoietic stem cell migration | 2 | 0.021682636 |
| BP | GO:0045963~negative regulation of dopamine metabolic process | 2 | 0.021682636 |
| BP | GO:0002540~leukotriene production involved in inflammatory response | 2 | 0.021682636 |
| BP | GO:0002477~antigen processing and presentation of exogenous peptide antigen via MHC class Ib | 2 | 0.021682636 |
| BP | GO:1904093~negative regulation of autophagic cell death | 2 | 0.021682636 |
| BP | GO:0001805~positive regulation of type III hypersensitivity | 2 | 0.021682636 |
| BP | GO:0002587~negative regulation of antigen processing and presentation of peptide antigen via MHC class II | 2 | 0.021682636 |
| BP | GO:0002491~antigen processing and presentation of endogenous peptide antigen via MHC class II | 2 | 0.021682636 |
| CC | GO:1904724~tertiary granule lumen | 4 | 0.021770516 |
| MF | GO:0004051~arachidonate 5-lipoxygenase activity | 2 | 0.021875078 |
| CC | GO:0005942~phosphatidylinositol 3-kinase complex | 3 | 0.023400929 |
| BP | GO:0002755~MyD88-dependent toll-like receptor signaling pathway | 3 | 0.023671735 |
| BP | GO:0046718~viral entry into host cell | 5 | 0.023902683 |
| CC | GO:0031410~cytoplasmic vesicle | 9 | 0.024130343 |
| CC | GO:0032587~ruffle membrane | 5 | 0.024210675 |
| BP | GO:0050730~regulation of peptidyl-tyrosine phosphorylation | 3 | 0.025742838 |
| MF | GO:0043274~phospholipase binding | 3 | 0.026168149 |
| BP | GO:0030031~cell projection assembly | 3 | 0.027884676 |
| MF | GO:0033691~sialic acid binding | 3 | 0.028343633 |
| CC | GO:0101003~ficolin-1-rich granule membrane | 4 | 0.028497334 |
| CC | GO:0035580~specific granule lumen | 4 | 0.029713688 |
| BP | GO:0032693~negative regulation of interleukin-10 production | 3 | 0.030095476 |
| BP | GO:0032727~positive regulation of interferon-alpha production | 3 | 0.030095476 |
| CC | GO:0033001~Fc-gamma receptor III complex | 2 | 0.032147049 |
| BP | GO:1903615~positive regulation of protein tyrosine phosphatase activity | 2 | 0.032347837 |
| BP | GO:0002469~myeloid dendritic cell antigen processing and presentation | 2 | 0.032347837 |
| BP | GO:0046633~alpha-beta T cell proliferation | 2 | 0.032347837 |
| BP | GO:0071727~cellular response to triacyl bacterial lipopeptide | 2 | 0.032347837 |
| BP | GO:0045058~T cell selection | 2 | 0.032347837 |
| BP | GO:0001766~membrane raft polarization | 2 | 0.032347837 |
| BP | GO:1905114~cell surface receptor signaling pathway involved in cell-cell signaling | 2 | 0.032347837 |
| BP | GO:2000363~positive regulation of prostaglandin-E synthase activity | 2 | 0.032347837 |
| BP | GO:0001788~antibody-dependent cellular cytotoxicity | 2 | 0.032347837 |
| BP | GO:2001190~positive regulation of T cell activation via T cell receptor contact with antigen bound to MHC molecule on antigen presenting cell | 2 | 0.032347837 |
| BP | GO:2001189~negative regulation of T cell activation via T cell receptor contact with antigen bound to MHC molecule on antigen presenting cell | 2 | 0.032347837 |
| BP | GO:0002286~T cell activation involved in immune response | 3 | 0.032373496 |
| BP | GO:0045954~positive regulation of natural killer cell mediated cytotoxicity | 3 | 0.032373496 |
| BP | GO:0043410~positive regulation of MAPK cascade | 6 | 0.032476566 |
| MF | GO:0030369~ICAM-3 receptor activity | 2 | 0.032633366 |
| BP | GO:0050860~negative regulation of T cell receptor signaling pathway | 3 | 0.034717023 |
| BP | GO:0034341~response to interferon-gamma | 3 | 0.034717023 |
| CC | GO:0034774~secretory granule lumen | 5 | 0.036531931 |
| CC | GO:0008305~integrin complex | 3 | 0.036708576 |
| BP | GO:0042832~defense response to protozoan | 3 | 0.037124372 |
| BP | GO:0070269~pyroptosis | 3 | 0.037124372 |
| BP | GO:0007200~phospholipase C-activating G-protein coupled receptor signaling pathway | 4 | 0.039579767 |
| BP | GO:0051602~response to electrical stimulus | 3 | 0.039593887 |
| BP | GO:0007202~activation of phospholipase C activity | 3 | 0.042123943 |
| BP | GO:0001817~regulation of cytokine production | 4 | 0.04250774 |
| CC | GO:0030526~granulocyte macrophage colony-stimulating factor receptor complex | 2 | 0.042632436 |
| BP | GO:0045622~regulation of T-helper cell differentiation | 2 | 0.042897316 |
| BP | GO:0035585~calcium-mediated signaling using extracellular calcium source | 2 | 0.042897316 |
| BP | GO:0002724~regulation of T cell cytokine production | 2 | 0.042897316 |
| BP | GO:0038156~interleukin-3-mediated signaling pathway | 2 | 0.042897316 |
| BP | GO:0014005~microglia development | 2 | 0.042897316 |
| BP | GO:0045728~respiratory burst after phagocytosis | 2 | 0.042897316 |
| BP | GO:2000516~positive regulation of CD4-positive, alpha-beta T cell activation | 2 | 0.042897316 |
| BP | GO:0038157~granulocyte-macrophage colony-stimulating factor signaling pathway | 2 | 0.042897316 |
| BP | GO:2000566~positive regulation of CD8-positive, alpha-beta T cell proliferation | 2 | 0.042897316 |
| BP | GO:0002316~follicular B cell differentiation | 2 | 0.042897316 |
| BP | GO:0030866~cortical actin cytoskeleton organization | 3 | 0.044712938 |
| BP | GO:0007498~mesoderm development | 3 | 0.044712938 |
| CC | GO:0009898~cytoplasmic side of plasma membrane | 4 | 0.046395688 |
| CC | GO:0042995~cell projection | 6 | 0.048295278 |

Legend: GO, gene ontology; BP, biological process; MF, molecular function; CC, cellular component.
